# Statin-induced lipid carrier stress reveals a conserved vulnerability in β-lactam-resistant Gram-positive bacteria

**DOI:** 10.1038/s41467-026-75729-8

**Published:** 2026-07-20

**Authors:** Gabriel Torrens, Sean W. Bisset, Maria López-Bravo, Anders F. Johansson, Daniel Lopez, Felipe Cava

**Affiliations:** 1 Department of Molecular Biology, Umeå University, Umeå, Sweden; 2 National Centre for Biotechnology, Spanish National Research Council (CNB-CSIC), Madrid, Spain; 3 Department of Clinical Microbiology, Umeå University, Umeå, Sweden; 4 The Laboratory for Molecular Infection Medicine Sweden (MIMS), Umeå Center for Microbial Research (UCMR), Science for Life Laboratory (SciLifeLab), Umeå, Sweden

**Keywords:** Antimicrobial resistance, Antibacterial drug resistance

## Abstract

Methicillin-resistant *Staphylococcus aureus* resists β-lactam antibiotics through the allosteric transpeptidase penicillin-binding protein 2a, which operates within staphyloxanthin-rich membrane microdomains. Statins restore susceptibility by disrupting these microdomains and impairing penicillin-binding protein 2a oligomerization, but the mechanisms enabling resistance to this resensitization remain unclear. Here we show, using evolution experiments in strains lacking a functional staphyloxanthin pathway, that mutations in *gdpP*, a regulator of cyclic di-adenosine monophosphate signaling, are the predominant route for restoring oxacillin resistance during membrane microdomain disruption. This adaptation is blocked by simvastatin, revealing a synthetic lethal interaction. Mechanistically, simvastatin inhibits the mevalonate pathway, depleting the essential lipid carrier undecaprenyl phosphate and exacerbating peptidoglycan precursor imbalance, an effect phenocopied by lipid carrier–targeting antibiotics such as bacitracin. Although compensatory mutations can restore resistance, they impose a fitness cost in vivo. Importantly, this vulnerability extends to *Streptococcus pneumoniae*, revealing a conserved strategy to overcome β-lactam resistance in Gram-positive pathogens.

## Abstract (web summary)

This study reveals that targeting a key lipid carrier can resensitize resistant Gram-positive bacteria to β-lactam antibiotics while making it much harder for them to evolve escape mutations due to high fitness costs.

## Introduction

The rise of antimicrobial resistance threatens the future of infection treatment, making it crucial to understand the molecular mechanisms that enable bacteria to evade antibiotics. *Staphylococcus aureus* is a Gram-positive bacterium commonly found on the skin and nostrils of healthy people. However, when this organism moves to another body niche such as the bloodstream, lungs, or heart valves, it can cause serious infections including sepsis, pneumonia, endocarditis, and osteomyelitis^1^. The methicillin-resistant variant (MRSA) has become a global threat due to its resistance to β-lactam antibiotics such as methicillin or oxacillin (OXA)^2^. MRSA infections can occur in the community or in healthcare settings. People who have weakened immune systems, have undergone invasive medical procedures, or are in close contact with others in crowded settings are at a higher risk of MRSA infection.

Although most MRSA infections remain treatable, antimicrobial resistance complicates therapy and, particularly in invasive or deep-seated infections, effective management often requires surgical removal of infected tissue in addition to antibiotic treatment^3^.

MRSA strains use multiple mechanisms to resist β-lactam antibiotics, including the production of β-lactamases (*blaZ*)^4^. However, the main resistance mechanism is the modification of penicillin-binding proteins (PBPs)^5^. PBPs are essential enzymes that catalyze the final steps of peptidoglycan (PG) synthesis, a critical process for bacterial cell-wall integrity. PG is a polymer composed of alternating N-acetylglucosamine (GlcNAc) and N-acetylmuramic acid (MurNAc) residues, crosslinked by short peptide chains. PBPs mediate transpeptidation, the enzymatic cross-linking of these peptide moieties, forming a mesh-like structure that provides mechanical strength and shape to the bacterial cell. As PG integrity is essential for bacterial survival, PBPs are key targets for antibiotics^6^. β-lactam antibiotics inhibit PBPs, preventing the bacteria from synthesizing a strong cell wall, which ultimately leads to cell lysis^4^. Most MRSA strains encode an alternative PBP (PBP2a), encoded by the locus *mecA*. PBP2a is allosterically regulated; its active site is inaccessible to β-lactams, so these antibiotics cannot efficiently acylate the catalytic serine, conferring resistance^7,8^. The *mecA* gene is carried on a mobile genetic element called the staphylococcal chromosomal cassette *mec* (SCC*mec*), which can be transferred between bacteria via transduction and, under specific conditions, through plasmid-mediated conjugation, contributing to the spread of MRSA and β‑lactam resistance^9^. Given that PBP2a poses a significant challenge to treating MRSA infections, there is a constant need for alternative treatments^10^.

**Fig. 1:**
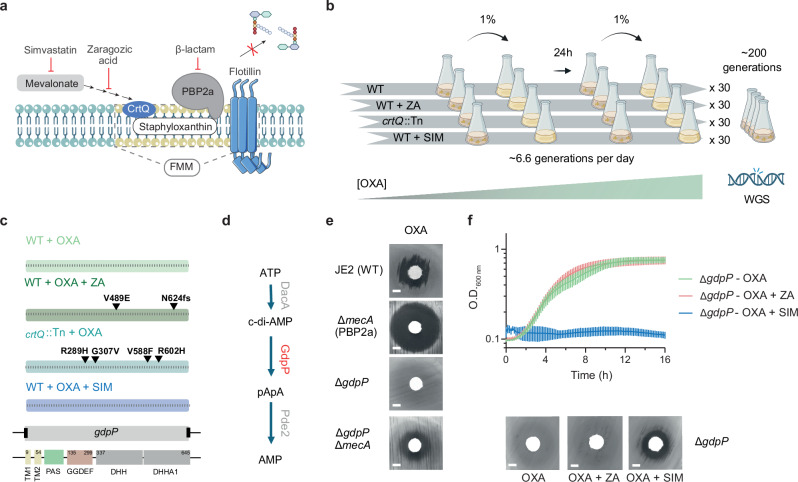
Statins modulate β-lactam susceptibility and evolutionary dynamics in *S. aureus* via functional membrane microdomains (FMM) stability, c-di-AMP signaling, and *gdpP*-mediated mechanisms. **a** Schematic illustration of the MRSA response to β-lactams and statins. Under β-lactam exposure, functional membrane microdomain (FMM) integrity and PBP2a-mediated transpeptidation are preserved, enabling bacterial survival. In contrast, statin treatment (e.g., simvastatin [SIM] or zaragozic acid [ZA]) inhibits staphyloxanthin biosynthesis, resulting in FMM destabilization, impaired PBP2a function, and ultimately MRSA cell death. **b** Schematic workflow of the long-term evolution experiment (LTEE), showing all genetic backgrounds and conditions for each line up to 200 generations, followed by sequencing. **c** LTEE sequencing results showing the *gdpP* gene (655 aa) affected by single-nucleotide polymorphisms (SNPs) or frameshifts (fs), relative to the reference sequence, after 200 generations of WT (LAC) exposed to oxacillin (OXA), with or without ZA or SIM, and the *crtQ::*Tn mutant (4,4′-diaponeurosporenoate glycosyltransferase mutant, which inhibits staphyloxanthin formation, from the Nebraska Transposon Mutant Library). Detailed changes are provided in Supplementary Data 1. **d** Overview of the c-di-AMP synthesis and degradation pathway in *S. aureus*. The cyclase DacA synthesizes c-di-AMP from ATP. GdpP phosphodiesterase (marked in red) linearizes c-di-AMP into phosphoadenylyl adenosine (pApA), which is then cleaved to AMP by the phosphatase Pde2. **e** Disk diffusion assay results with 5 µg/disk OXA, illustrating differences in antibiotic susceptibility among the strains (scale bar: 10 mm). **f** Growth curves of the *gdpP* mutant strain treated with or without 25 µM statins in the presence of 16 µg/mL of OXA (top). Data are mean ± standard deviation from *n* = 3 independent biological replicates. Disk diffusion assay of OXA (5 µg/disk) and statins (25 nmol/disk) using the *gdpP* mutant strain (scale bar: 10 mm) (bottom). **a** and **b** were created in BioRender: https://BioRender.com/v8m4cbb and https://BioRender.com/ttsmp1i. Source data are provided as a Source Data file.

Recent studies show that cholesterol-lowering drugs, including statins and the polyketide-like natural product zaragozic acid (ZA), can act as promising agents against MRSA^11^. Although ZA is not a statin in chemical terms, it targets the same pathway, justifying its designation as a squalestatin. For clarity, we refer to both classes collectively as statins throughout this work. Beyond their lipid-lowering effects, these drugs exhibit antibacterial properties, including disruption of bacterial membranes and interference with virulence factor expression^12^. Notably, retrospective cohort studies suggest that statins can enhance the efficacy of antibiotics, even restoring the activity of β-lactams, which are typically ineffective against MRSA^13^. This potentiation is thought to result from their ability to disrupt functional membrane microdomains (FMM)—specialized regions of the bacterial membrane enriched in specific lipids and proteins, analogous to lipid rafts in eukaryotic cells^11,14^. While FMMs contribute to membrane organization, their role extends to key cellular processes such as signaling, protein trafficking, and antibiotic resistance^14^. In MRSA, FMMs are enriched in staphyloxanthin lipids and proteins like flotillin, which facilitate PBP2a oligomerization, a critical step for β-lactam resistance^11,14^. Statins have been shown to impair staphyloxanthin synthesis, thereby dismantling FMMs and disrupting PBP2a oligomerization and function—ultimately restoring MRSA susceptibility to β-lactams (Fig. 1a)^11,15,16^. For instance, ZA significantly lowers the minimum inhibitory concentration (MIC) of OXA required to inhibit MRSA growth^11^. While these findings are promising, most research on statins’ antibacterial potential has focused on their impact on PBP2a, potentially overlooking *mecA*-independent mechanisms that may also contribute to MRSA resensitization.

Here, we show how MRSA regains resistance under conditions that destabilize FMMs. Using experimental evolution, we uncovered statin-specific adaptation pathways, with *gdpP* mutations emerging as a dominant mechanism to restore β-lactam resistance via cyclic di-adenosine monophosphate (c-di-AMP) signaling. However, this resistance pathway fails when MRSA is treated with simvastatin (SIM), a statin that blocks the mevalonate pathway and stresses the cell wall’s lipid carrier. This genetic–drug interaction disrupts cell-wall homeostasis and resensitizes resistant strains to β-lactams. Although compensatory mutations can restore resistance, they come at a fitness cost in vivo. We observed the same vulnerability in *Streptococcus pneumoniae*, suggesting a broad therapeutic opportunity. Together, our findings highlight the potential of undecaprenyl phosphate (C_55_-P) inhibitors as β-lactam adjuvants against MRSA and other clinically relevant Gram-positive pathogens.

## Results

### Statins drive divergent MRSA resistance pathways

Statins resensitize MRSA to OXA by disrupting FMMs, which affects PBP2a’s correct folding and oligomerization^11,14^. We hypothesized that long-term evolution experiments (LTEE) with MRSA strains lacking a functional staphyloxanthin pathway could reveal alternative, FMM-independent mechanisms for restoring PBP2a activity. To investigate this, we exposed the MRSA wild-type (WT) USA300 LAC strain, with or without statins, to increasing OXA concentrations. We used ZA and SIM, which inhibit different steps in staphyloxanthin synthesis (Fig. 1a, b). As a positive control for FMM deficiency, we included a *crtQ* mutant, which affects the final step of the staphyloxanthin pathway. This mutation increased sensitivity to OXA without affecting growth under standard conditions (Fig. 1a, Supplementary Fig. 1a, b). After 14 and 28 days, some lines gave rise to clones with significantly increased OXA MICs (Supplementary Fig. 1c). In contrast, those that showed no MIC changes after 200 generations were excluded from further analysis (Supplementary Fig. 1a–c).

Whole-genome sequencing (WGS) of evolved MRSA populations revealed a diverse mutational landscape, including recurrent *rpoB/rpoC* substitutions in some ZA- and *crtQ*-adapted lineages. Additional variants were also identified, including mutations in *norC* and *apt* in OXA/ZA-adapted populations and *clpX* in a SIM-adapted lineage, likely reflecting broader adaptive responses to β-lactam or statin pressure, although their individual contributions remain to be defined. Among these, *gdpP*—encoding a c-di-AMP phosphodiesterase—emerged as the most consistently selected target across multiple conditions (Fig. 1c, d, Supplementary Fig. 1d, e, Supplementary Data 1), consistent with its increasing prevalence in clinical MRSA isolates^17^ and its association with elevated β-lactam resistance even in *mecA*-negative strains^18^. Notably, most evolved *gdpP* variants were hypomorphic alleles, suggesting that the phenotype arises from elevated c-di-AMP levels rather than loss of GdpP per se.

Importantly, in contrast to ZA and *crtQ*-defective conditions, SIM-treated cultures did not select *gdpP* mutations (Fig. 1c, Supplementary Fig. 1d). Instead, several lineages carried the same *ftsK* A665G variant (Supplementary Fig. 1d), likely arising early or already present in the founding population. Although the contribution to SIM/OXA adaptation of these variants remains unclear, the absence of *gdpP* mutations indicates that SIM does not select for the GdpP/c-di-AMP resistance pathway, suggesting a distinct selective constraint.

The repeated recovery of *gdpP* mutations across independent lineages—often in the absence of *rpoB/rpoC* changes—and their selection under ZA and *crtQ*-defective conditions but not under SIM prompted us to focus on *gdpP* mechanistically, as this distinct selection pattern pointed to a specific and previously underappreciated link between c-di-AMP signaling, β-lactam resistance, and PG homeostasis.

To validate our LTEE results, we generated a clean *gdpP* in-frame deletion in *S. aureus* JE2, avoiding the LAC strain due to its hyper-resistant plasmid^19^. As previously reported^20,21^, Δ*gdpP* cells had a mild growth defect without antibiotics (Supplementary Fig. 2a); they were smaller (Supplementary Fig. 2b, c) and showed high-level OXA resistance (MIC ~ 256 µg/mL) (Supplementary Fig. 2d). Complementation restored WT phenotypes (Supplementary Fig. 2a–d). Notably, the resistance effect was independent of PBP2a, as Δ*gdpP* mutations emerged in FMM-impaired strains and increased OXA MIC 8-fold in both WT and Δ*mecA* backgrounds (Fig. 1e, Supplementary Fig. 2e). Interestingly, while sublethal concentrations of both ZA and SIM enhanced OXA sensitivity in WT MRSA (Supplementary Fig. 2f–h), only SIM—not ZA—restored OXA sensitivity in the *gdpP* mutant (Fig. 1f, Supplementary Fig. 2i), consistent with our evolution experiment results. To quantify the OXA–SIM interaction, we performed checkerboard assays in WT, Δ*gdpP*, and *crtQ*::Tn strains. SIM alone inhibited growth only at high concentrations (200 µM), whereas subinhibitory levels potentiated OXA. Fractional inhibitory concentration (FIC) and isobologram analyses revealed synergy in all backgrounds, with a markedly stronger interaction in Δ*gdpP* (Supplementary Fig. 3a–c). Together, these results uncover a specific antagonism between SIM and GdpP-mediated resistance, revealing a vulnerability that can be exploited to restore β-lactam sensitivity in MRSA, including *gdpP* mutant strains commonly found in highly resistant clinical isolates.

### Peptidoglycan precursor deficiency underlies the oxacillin-dependent synthetic lethality between simvastatin and GdpP loss

**Fig. 2:**
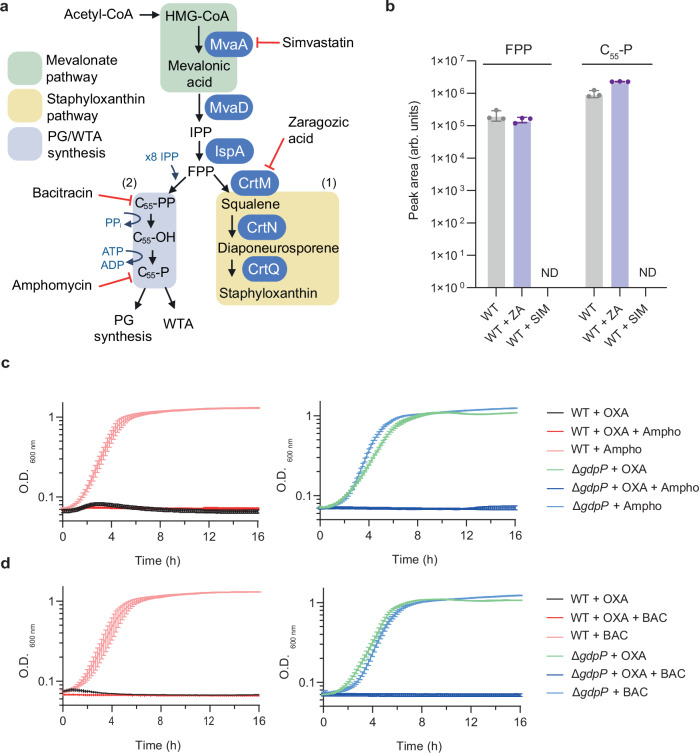
Impact of statins and antibiotic peptides on mevalonate-derived pathways and growth in *S. aureus.* **a** Schematic representation of the mevalonate pathway starting from acetyl-CoA, leading to the formation of isopentenyl pyrophosphate (IPP) and farnesyl pyrophosphate (FPP). From FPP, two distinct biosynthetic pathways diverge: (1) the staphyloxanthin pathway, responsible for the production of carotenoid pigments in *Staphylococcus* species, and (2) the undecaprenyl phosphate (C_55_-P) pathway, which provides essential lipid carriers required for peptidoglycan (PG) synthesis and wall teichoic acids (WTA) biosynthesis in bacterial cell-wall formation. Sites of inhibition by statins (simvastatin targeting HMG-CoA reductase MvaA and zaragozic acid targeting squalene synthase CrtM) and by antibiotic peptides are indicated. **b** Comparative analysis of key isoprenoid intermediates FPP and undecaprenyl phosphate (C_55_-P) in WT strain in the presence or absence of statins (25 µM). Peak areas were measured using UPLC-UV absorbance at 210 nm and expressed in arbitrary units (arb. units). ND, not detected. No statistical test was performed because FPP and C55-P were below the assay’s limit of detection following simvastatin treatment. Growth curves of WT and *gdpP* mutant strains treated with or without oxacillin (OXA, 16 µg/mL) with or without amphomycin (Ampho, 40 µg/mL) (**c**) or bacitracin (BAC, 30 µg/mL) (**d**). For all panels, data are presented as mean ± standard deviation from *n* = 3 independent biological replicates, except for WT + amphomycin, Δ*gdpP* + OXA, and Δ*gdpP* + amphomycin, for which *n* = 4 independent biological replicates. SIM simvastatin, ZA zaragozic acid. (**a**) was created in BioRender: https://BioRender.com/l3miaiz. Source data are provided as a Source Data file.

How does SIM overcome the *gdpP*-mediated OXA resistance pathway in MRSA? To answer this, we compared the effects of ZA and SIM as FMM inhibitors. Both statins eliminate the yellow staphyloxanthin pigment in *S. aureus*, a process that starts from the intermediate farnesyl pyrophosphate (FPP) (Fig. 2a, Supplementary Fig. 4a). FPP is also crucial for producing C_55_-P—the lipid carrier required for PG transport across the membrane^22^. While ZA acts late in this pathway, SIM blocks an earlier step, halting the synthesis of both FPP and C_55_-P (Fig. 2a). This broader inhibition likely explains why SIM, but not ZA, can break *gdpP*-driven resistance, suggesting that by depleting the lipid carrier pool, SIM disrupts cell-wall homeostasis and exposes a vulnerability in resistant MRSA strains.

To investigate this, we quantified FPP and C_55_-P levels in statin-treated MRSA using ultra-performance liquid chromatography (UPLC). Interestingly, while FPP levels remained unchanged with ZA, this compound led to C_55_-P accumulation, whereas SIM reduced both metabolites to undetectable levels (Fig. 2b, Supplementary Fig. 4b, c). These results suggest that *gdpP* mutations were preferentially selected in conditions involving ZA or *crtQ* disruption due to their preservation of the C_55_-P pool, which is critical for PG synthesis. Supporting this, we found that amphomycin and bacitracin, antibiotics that inhibit C_55_-P recycling^22^ (Figs. 2a, 2c, d), mimicked the synergistic effect of SIM in the *gdpP* mutant. Collectively, these results reveal that SIM’s targeted inhibition of early steps in PG biogenesis creates a synthetic lethal interaction with *gdpP* mutations, underscoring the central importance of the C_55_-P lipid carrier in safeguarding cell-wall integrity under antibiotic stress.

**Fig. 3:**
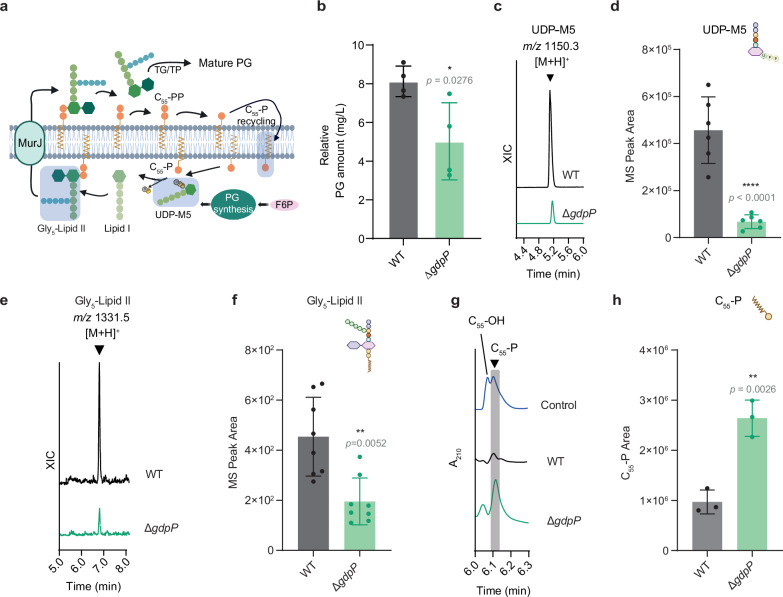
Cell wall biosynthesis intermediates in the *gdpP* mutant and WT strains. **a** Schematic overview of peptidoglycan (PG) biosynthesis starting from fructose-6-phosphate (F6P), leading to the formation of UDP-MurNAc pentapeptide (UDP-M5) and subsequent synthesis of Lipid II through its linkage to undecaprenyl phosphate (C_55_-P). Lipid II is then translocated across the cytoplasmic membrane and incorporated into mature PG by transglycosylase (TG) and transpeptidase (TP) activities. **b** Relative PG amount determined by weighing freeze-dried sacculi in WT and *gdpP* mutant strains. Data are mean ± standard deviation from *n* = 4 independent biological replicates. **c** Extracted ion chromatogram (XIC) of UDP-M5 (*m/z* 1150.3 [M + H]^+^) obtained by LC-MS. **d** UDP-M5 levels based on MS peak area. Data are mean ± standard deviation from *n* = 6 independent biological replicates. **e** Extracted ion chromatogram (XIC) of Gly_5_-Lipid II (*m/z* 1331.5 [M + H]^+^) obtained by LC-MS. **f** Gly_5_-Lipid II levels based on MS peak area. Data are mean ± standard deviation from *n* = 8 independent biological replicates. **g** UPLC-UV chromatograms (210 nm absorbance) showing C_55_-P levels in the *gdpP* mutant relative to the WT strain. Standards of 200 nmol C_55_-OH (undecaprenol) and C_55_-P (undecaprenyl phosphate) are included as controls. **h** Corresponding C_55_-P peak area quantification. Data are mean ± standard deviation from *n* = 3 independent biological replicates. Statistical significance was conducted using a Student’s *t*-test (unpaired, two-tailed) with significance indicated as *p < 0.05; **p < 0.01; ***p < 0.001; ****p < 0.0001. Panel a was created in BioRender: https://BioRender.com/n046fkw. Source data are provided as a Source Data file.

To determine if *gdpP* disruption increases SIM sensitivity by impairing PG synthesis (Fig. 3a), we analyzed muropeptide profiles using liquid chromatography–mass spectrometry (LC-MS). In line with previous reports^20,23^, the PG profile of the *gdpP*-disrupted strain closely mirrored that of the WT (Supplementary Fig. 5a). However, quantifications revealed a significant reduction in total PG content per dry cell mass in the mutant (Fig. 3b), reflecting impaired biosynthetic capacity. This suggests that *gdpP* loss compromises early PG biosynthesis, making the strain especially vulnerable to further inhibition by SIM.

To explore this further, we quantified the soluble PG precursor intermediate UDP-MurNAc-pentapeptide (UDP-M5). Strikingly, the *gdpP* mutant strain exhibited a five-fold reduction in UDP-M5 levels (Fig. 3c, d), a phenotype also observed in *mecA* and *crtQ* backgrounds (Supplementary Fig. 5b). UDP-M5 combines with the lipid carrier C_55_-P to form lipid I, which is then modified by the addition of GlcNAc and a pentaglycine bridge to generate Gly_5_-Lipid II—the final PG precursor in *S. aureus* (Fig. 3a)^24^. Consistent with impaired precursor synthesis, we detected a significant reduction in Gly_5_-Lipid II (Fig. 3e, f) and a corresponding accumulation of free C_55_-P in the *gdpP* mutant compared to the WT strain (Fig. 3g, h). These findings indicate that GdpP plays a critical role in maintaining balanced PG precursor flux, and its disruption leads to bottlenecks that sensitize cells to further inhibition by compounds like SIM, which deplete the lipid carrier pool.

### GdpP disruption impairs multiple stages of PG precursor synthesis

To pinpoint the cause of reduced UDP-M5 levels in the *gdpP* mutant, we first overproduced MurA—the initial enzyme in the PG biosynthetic pathway and a key regulator of precursor abundance (Supplementary Fig. 6a)^25,26^. Despite achieving ~5-fold overproduction of MurA (Supplementary Fig. 6b), only the WT strain showed a modest but statistically significant increase in PG content, whereas PG levels in the *gdpP* mutant remained unchanged (Supplementary Fig. 6c, d). OXA resistance was similarly unaffected in both backgrounds (Supplementary Fig. 6c, d), indicating that MurA activity is not the limiting factor in the mutant and suggesting that the UDP-M5 deficit stems from upstream substrate limitations. To test this, we supplemented cultures with D-(-)-fructose, a precursor of fructose-6-phosphate—the metabolic entry point for UDP-GlcNAc biosynthesis. However, this intervention failed to restore PG levels or OXA sensitivity in the *gdpP* mutant (Supplementary Fig. 6e, f), ruling out fructose-6-phosphate limitation as the underlying cause.

**Fig. 4:**
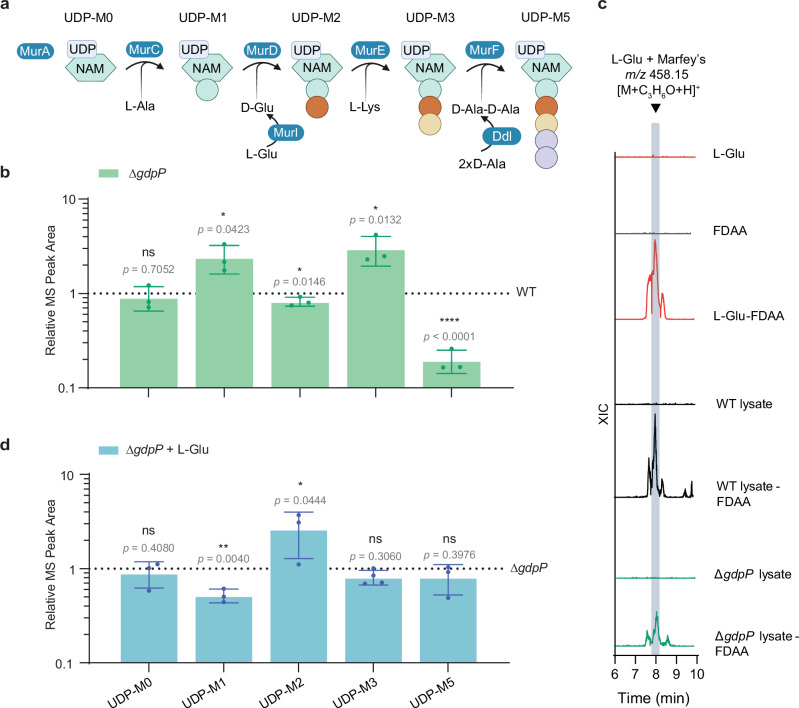
Deficient intracellular L-glutamate in *gdpP* mutants and its effects on Mur pathway metabolite levels. **a** Diagram illustrating the Mur pathway for UDP-MurNAc-pentapeptide (UDP-M5) biosynthesis, showing the sequential addition of amino acids to UDP-N-acetylmuramic acid (UDP-MurNAc); Amino acids L-alanine (L-Ala), D-glutamate (D-Glu), L-lysine (L-Lys), 2× D-alanine (D-Ala), UDP: uridine diphosphate, NAM: MurNAc. **b** Levels of Mur pathway metabolites in the *gdpP* mutant relative to WT, based on relative MS peak areas. Data are mean ± standard deviation from *n* = 3 independent biological replicates. **c** Representative extracted ion chromatograms (XICs) of L-Glu (*m/z* 458.15 [M + C₃H₆O + H]^+^) from bacterial lysates with and without derivatization using Marfey’s reagent (FDAA; 1-fluoro-2-4-dinitrophenyl-5-L-alanine amide). **d** Levels of Mur pathway metabolites in the *gdpP* mutant supplemented with 10 mM L-glutamate (L-Glu), relative to the *gdpP* mutant without supplementation, based on relative MS peak areas. Data are presented as mean ± standard deviation from *n* = 3 independent biological replicates, except for UDP-M3, for which *n* = 4 independent biological replicates. Statistical significance was conducted using a Student’s *t*-test (unpaired, two-tailed) with significance indicated as *p < 0.05; **p < 0.01; ***p < 0.001; ****p < 0.0001. ns, not significant. Panel a was created in BioRender: https://BioRender.com/d0bpzzi. Source data are provided as a Source Data file.

We next quantified all soluble intermediates from UDP-MurNAc (UDP-M0) to UDP-M5 (Fig. 4a) to map the metabolic bottlenecks. The *gdpP* mutant exhibited clear accumulation of UDP-M1 and UDP-M3, along with reduced levels of UDP-M2 and UDP-M5 (Fig. 4b, Supplementary Fig. 7a), indicating multiple disruptions along the Mur pathway. To further dissect these metabolic disruptions, we hypothesized that the accumulation of UDP-M1 in the *gdpP* mutant could be due to limited availability of L-glutamate (L-Glu), a critical substrate for the MurD reaction and the most abundant free amino acid in the cytoplasm^27^. Given that *gdpP* mutants exhibit elevated ppGpp levels^23,28^ —a hallmark of the stringent response to nutrient stress—we tested whether L-Glu levels were affected. Using Marfey’s derivatization and LC-MS, we found that free L-Glu was significantly reduced in the *gdpP* mutant compared to the WT (Fig. 4c, Supplementary Fig. 7b), supporting the idea that L-Glu limitation impairs the MurD step (Fig. 4a).

In parallel, we also observed accumulation of UDP-M3, suggesting a second bottleneck at the Ddl ligase step. Previous studies in *Listeria monocytogenes* have shown that high c-di-AMP levels and reduced potassium availability can impair Ddl activity^29^. To test whether L-Glu supplementation could overcome these defects, we added 10 mM L-Glu to the *gdpP* mutant. This intervention restored UDP-M2 levels but had no effect on UDP-M5 (Fig. 4d, Supplementary Fig. 7a), indicating that while L-Glu supplementation rescues the MurD step, the Ddl bottleneck persists—likely due to impaired potassium-dependent enzyme activity.

Together, these findings demonstrate that *gdpP* disruption impairs PG precursor synthesis at multiple points, creating vulnerabilities that are further amplified by SIM-induced depletion of the lipid carrier pool. This multifaceted disruption ultimately compromises cell-wall integrity and sensitizes MRSA to antibiotic stress.

### Suppressor mutations in RNA polymerase and PG remodeling rescue oxacillin resistance in *gdpP*-deficient strains treated with simvastatin

**Fig. 5:**
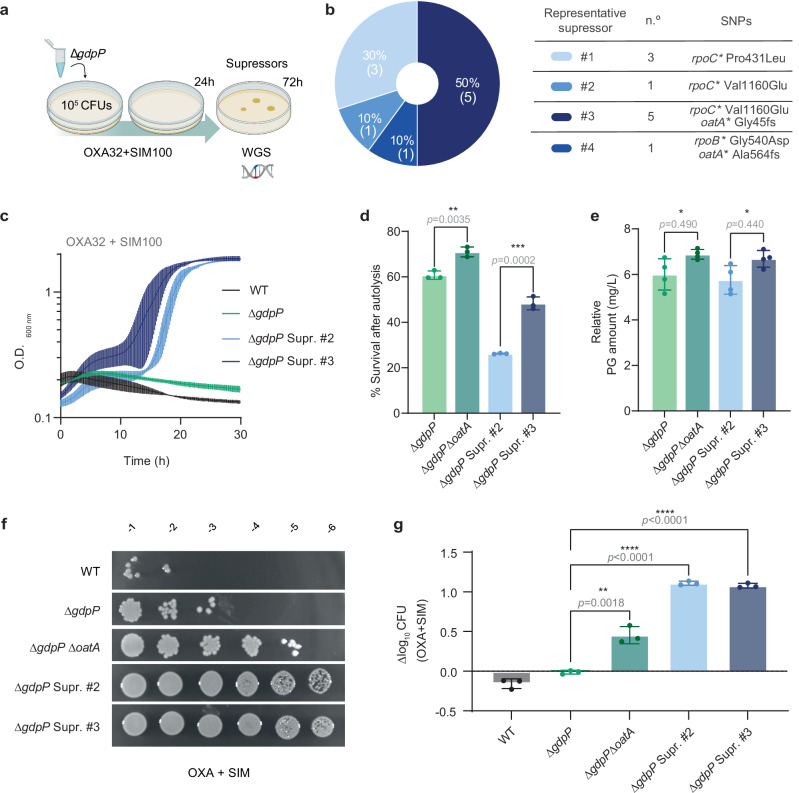
Phenotypic and genotypic characterization of *gdpP* suppressor mutants under oxacillin and simvastatin stress. **a** Workflow for the generation of suppressor mutants in the *gdpP* strain under lethal concentrations of oxacillin (OXA; 32 µg/mL) and simvastatin (SIM; 100 µM) for WGS. **b** Identified suppressors and frequency in 10 isolates. **c** Growth curves of suppressor isolates #2 and #3 compared to their parental strain and WT oxacillin (OXA, 32 µg/mL) and simvastatin (SIM, 100 µM). **d** Autolysis assay showing CFU/mL after 240 min in the presence of 0.05% Triton X-100 in the *gdpP* mutant, *gdpP-oatA* double mutant, and the suppressor derivatives #2 and #3. **e** Peptidoglycan (PG) quantification by weighing freeze-dried sacculi of the *gdpP* mutant, *gdpP-oatA* double mutant, and the suppressor derivatives #2 and #3. **f** Spot assay after overnight growth in TSB containing 16 µg/mL oxacillin (OXA) and 25 µM simvastatin (SIM). **g** Relative survival after growth in TSB with 16 µg/mL OXA and 25 µM simvastatin (SIM), calculated as Δlog_10_ CFU (log_10_ CFU 24 h minus log_10_ CFU at 0 h; *n* = 3). For (**c**), (**d**), and (**g**), data are presented as mean ± standard deviation from *n* = 3 independent biological replicates, except for (**e**), for which *n* = 4 independent biological replicates. Statistical significance was conducted using a Student’s *t*-test (unpaired, two-tailed) with significance indicated as *p < 0.05; **p < 0.01; ***p < 0.001; ****p < 0.0001. (**a**) was created in BioRender: https://BioRender.com/mannhll. Source data are provided as a Source Data file.

While defects in PG precursor synthesis explain the OXA-dependent synthetic lethality of the *gdpP* mutation with SIM, they do not account for the strain’s regained resistance to OXA. To investigate this, we conducted a suppressor screen of the Δ*gdpP* background under SIM selection, aiming to uncover genetic interactions that bypass SIM’s resensitizing effect (Fig. 5a). All identified suppressor mutants (Fig. 5b) exhibited robust growth in tryptic soy broth (TSB) medium with OXA and SIM (Supplementary Fig. 8a–c), mirroring the parental Δ*gdpP* strain, but with significantly increased resistance to OXA despite SIM treatment (Fig. 5c, Supplementary Fig. 8b, c). These findings suggest that the suppressor mutations override SIM’s resensitizing effect, enabling MRSA to maintain high-level OXA resistance even when FMMs are compromised.

WGS revealed that all suppressor mutants carried mutations in either the β (RpoB G540D) or β′ (RpoC V1160E; P431L) subunits of the RNA polymerase (Fig. 5b). Like *gdpP*, mutations in *rpoB* and *rpoC* are commonly emerging potentiators of β-lactam resistance^30^. However, the distinct growth profiles of these mutants under SIM treatment suggest that their resistance mechanisms diverge from those of *gdpP*. Intriguingly, 50% of the suppressor isolates also carried frameshift mutations (G45fs; A564fs) in the MurNAc O-acetyltransferase OatA^31^ (Fig. 5b), resulting in significantly reduced PG O-acetylation compared to their parental strains (Supplementary Fig. 8d). Consistent with this, *oatA*-mutated suppressors showed increased sensitivity under combined lysozyme/tunicamycin challenge, with only minor effects observed under lysozyme alone (Supplementary Fig. 8e, f), consistent with the role of PG O-acetylation in cell-wall protection, particularly under conditions of cell envelope stress^31–35^.

To isolate the contribution of *oatA* mutations to OXA resistance, we compared two suppressor strains carrying identical *rpoC* mutations but differing in their *oatA* status. Suppressor #3 (mutated *oatA*) exhibited significantly higher OXA resistance than suppressor #2 (WT *oatA* allele) (Fig. 5c), indicating that *oatA* inactivation enhances resistance in the context of impaired PG biogenesis.

Because Atl, the major autolysin in *S. aureus*, preferentially targets O-acetylated PG^36^, we hypothesized that loss of OatA function reduces PG turnover, thereby stabilizing the cell wall under stress. Consistent with this, suppressor #3 showed pronounced resistance to Triton X-100–induced autolysis and accumulated more PG per cell than suppressor #2 (Fig. 5d, e). The strains did not differ in morphology (Supplementary Fig. 8g), indicating that the increased PG content reflects reduced autolytic degradation rather than changes in cell shape.

To test this directly, we introduced an *oatA* mutation into the Δ*gdpP* background, which recapitulated the suppressor phenotype: PG levels increased, autolysis was reduced, and growth improved under combined OXA and SIM treatment (Fig. 5d–g). This indicates that limiting PG turnover provides a compensatory advantage when cell-wall synthesis is impaired. Consistent with this, checkerboard assays of the Δ*gdpP* Δ*oatA* strain revealed a loss of synergy between OXA and SIM (FIC = 0.516; Supplementary Fig. 9a, b).

Together, these results support a model in which *oatA* mutations promote OXA resistance by reducing autolytic PG degradation, thereby reinforcing cell-wall integrity under conditions of impaired PG biosynthesis.

### Simvastatin as an in vivo adjuvant and the fitness cost of suppressor mutations

**Fig. 6:**
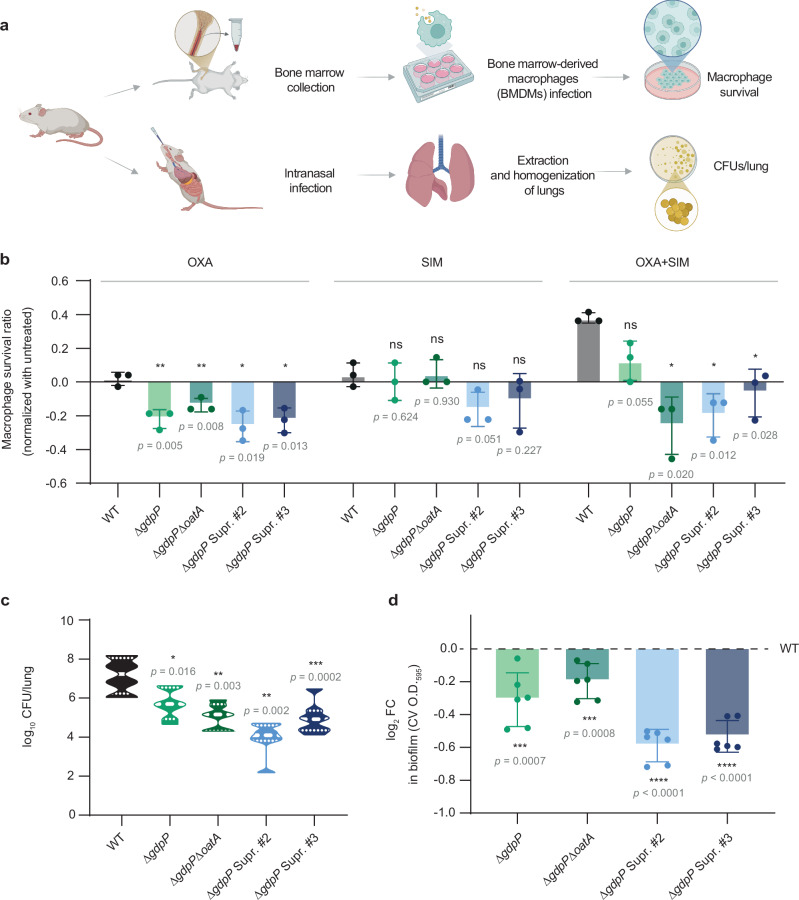
In vitro and in vivo infection outcomes and associated phenotypes across WT, parental, and suppressor strains. **a** Murine infection workflow. From a mouse, the schematic shows two branches: bone marrow extraction and differentiation into bone marrow–derived macrophages (BMDMs) followed by bacterial infection, and intranasal in vivo infection followed by lung extraction and homogenization to determine CFU per lung. **b** Log_2_ macrophage survival ratios normalized to the untreated condition, measured after infection of BMDMs at a 4:1 bacteria:macrophage ratio. Treatments compared include oxacillin (OXA, 250 µg/mL), simvastatin (SIM, 25 µM), or both. Data are mean ± standard deviation from *n* = 3 independent biological replicates. **c** Bacterial burden in mouse lungs (log_10_ CFU per lung) after intranasal infection with 2 × 10⁸ CFU per mouse. *n* = 4 for WT, *n* = 7 for Δ*gdpP,* and for Δ*gdpP* Supr. #2, *n* = 7 for Δ*gdpP* Supr. #3, and *n* = 6 for Δ*gdpP* Δ*oatA*. **d** Log_2_ fold change in biofilm biomass quantified by crystal violet (CV) staining (OD_595_). Data are mean ± standard deviation from *n* = 6 independent biological replicates. All panels include WT, suppressor mutants, and parental strains. Statistical analyses were performed using an unpaired *t*-test (two-tailed) for (**c**) and (**d**), and an unpaired *t*-test (two-tailed) with Welch’s correction for panel b. Significance was indicated as *p < 0.05; **p < 0.01; ***p < 0.001; ****p < 0.0001. ns, not significant. (**a**) was created in BioRender: https://BioRender.com/7azwgbx. Source data are provided as a Source Data file.

To assess the therapeutic potential of SIM as an in vivo adjuvant, we first examined its impact on MRSA infection in bone marrow–derived macrophages (Fig. 6a). In the presence of OXA, the WT strain was efficiently neutralized, resulting in only modest improvements in macrophage survival (Fig. 6b)—consistent with limited intracellular antibiotic activity^37^. In contrast, the Δ*gdpP* mutant was able to overcome OXA treatment and severely compromise macrophage viability (Fig. 6b). Remarkably, this heightened virulence was reversed by SIM (Fig. 6b), but not by ZA (Supplementary Fig. 10a), mirroring the synthetic lethality observed in vitro and highlighting SIM’s unique ability to restore antibiotic sensitivity. Suppressor mutants carrying compensatory mutations in *rpoB*/*C* and *oatA* continued to reduce macrophage survival even in the presence of SIM. This intermediate phenotype suggests that these mutations partially mitigate SIM-mediated lethality by conferring a modest fitness advantage.

To directly evaluate fitness, we tested each strain’s ability to infect and kill macrophages without antibiotics. The WT strain efficiently infected and killed macrophages, whereas all mutant strains showed impaired infectivity. The *ΔgdpP* Supr. #2 was most severely affected, with minimal infection capacity, while the *ΔgdpP* and Supr. #3 also displayed reduced infectivity, though to a lesser degree (Supplementary Fig. 10b).

We then validated these findings in vivo using a mouse model of intranasal infection (Fig. 6a). The WT strain established robust infections, reaching ~10⁸ colony-forming units (CFU)/g and causing clinical symptoms. In contrast, all mutant strains showed significantly reduced colonization: the *ΔgdpP* Supr. #2 mutant was nearly cleared, and the *ΔgdpP* strain reached only ~10⁶ CFU/g, with other mutants remaining below this threshold (Fig. 6c). Biofilm formation mirrored these trends: the *ΔgdpP* Supr. #2 and #3 showed a pronounced reduction in biofilm formation, while the Δ*gdpP* and Δ*gdpP* Δ*oatA* mutants also exhibited decreased biofilm capacity, though less severe (Fig. 6d), further supporting their impaired ability to colonize host tissue.

Together, these results demonstrate that SIM can effectively restore antibiotic sensitivity in highly resistant *gdpP* mutants during macrophage infection. Moreover, the mouse infection model reveals that while suppressor mutations can compensate under antibiotic stress, they impose a substantial fitness cost in the absence of treatment—limiting the pathogen’s ability to establish and sustain infection.

### C-di-AMP phosphodiesterase inhibition reveals conserved phenotypes in mevalonate-pathway bacteria

**Fig. 7:**
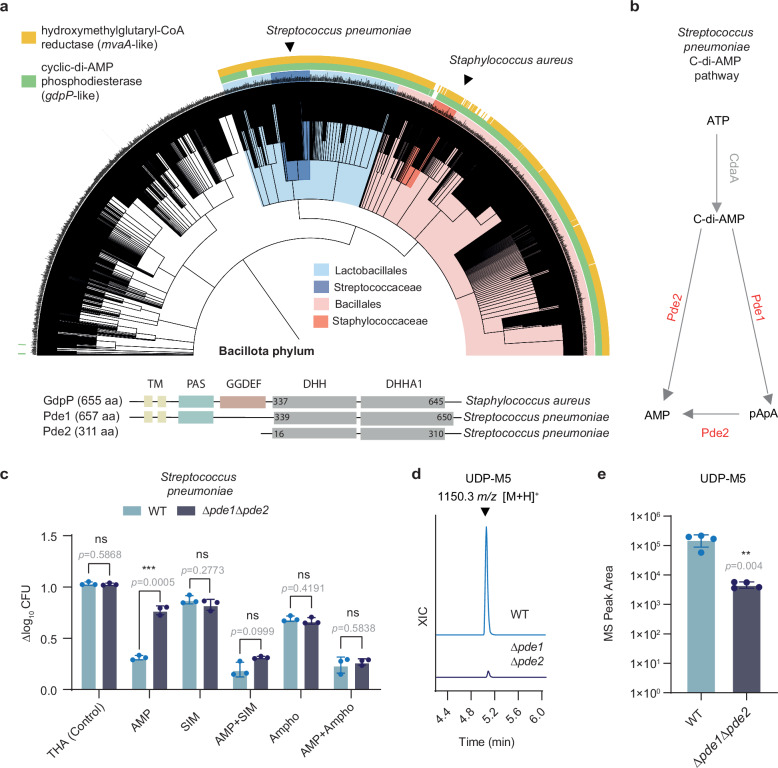
Conserved impact of phosphodiesterase deficiency on peptidoglycan metabolism across Bacillota and its modulation by C_55_-P inhibitors. **a** Phylogenetic tree of the phylum Bacillota highlighting orthologs of c-di-AMP phosphodiesterases (*gdpP*/*pde1*/*pde2*-like; OrthoDB group: 246344at91061, green) and hydroxymethylglutaryl-CoA reductase (*mvaA*-like; OrthoDB group: 340903at91061, yellow), the target of simvastatin. The two most extensive orders within Bacillota—Lactobacillales (blue) and Bacillales (red)—are indicated, with representative families (Streptococcaceae and Staphylococcaceae) highlighted. The positions of *Staphylococcus aureus* and *Streptococcus pneumoniae*, both of which possess the mevalonate pathway for isoprenoid biosynthesis, are indicated. The panel also includes a comparative analysis of the orthologous proteins GdpP, Pde1, and Pde2, along with alignments of their conserved domains. **b** Schematic representation of the c-di-AMP synthesis and degradation pathway in *S. pneumoniae*. The diadenylate cyclase CdaA (DacA in *S. aureus*) converts ATP into the second messenger c-di-AMP. The phosphodiesterase Pde1 hydrolyzes c-di-AMP into 5′-phosphoadenylyl adenosine (pApA), while Pde2 further cleaves pApA into two AMP molecules and can also directly degrade c-di-AMP. Phosphodiesterases are marked in red. **c** Relative survival of *S. pneumoniae* WT and the *pde1 pde2* double mutant expressed as Δlog_10_ CFU (log_10_ CFU at 24 h minus log_10_ CFU at 0 h). Treatments included ampicillin (AMP, 0.1 µg/mL), simvastatin (SIM, 25 µM), and amphomycin (Ampho, 40 µg/mL). Data are mean ± standard deviation from *n* = 3 independent biological replicates. **d** Extracted ion chromatogram (XIC) of UDP-M5 (*m/z* 1150.3 [M + H]^+^) obtained by LC-MS. **e** UDP-M5 levels based on the MS peak area. Data are mean ± standard deviation from *n* = 4 independent biological replicates. Statistical significance was conducted using a Student’s *t*-test (unpaired, two-tailed) with significance indicated as *p < 0.05; **p < 0.01; ***p < 0.001; ****p < 0.0001. ns, not significant. Source data are provided as a Source Data file.

To determine whether the OXA-dependent synthetic lethality between *gdpP* disruption and SIM extends beyond MRSA, we constructed a phylogenetic tree based on GdpP and MvaA—the mevalonate-pathway enzyme targeted by SIM (Fig. 7a). These two proteins are broadly distributed across the Bacillota phylum (formerly Firmicutes), motivating us to test whether the genetic interaction observed in MRSA extends to other species. We focused on *S. pneumoniae*, a key human pathogen of the taxonomic order Lactobacillales, which encodes two c-di-AMP phosphodiesterases^38^, *pde1* and *pde2* (Fig. 7b). Using WT and *pde1 pde2* double mutant strains, we observed that the double mutant, like the *gdpP* mutant in *S. aureus*, displayed increased resistance to ampicillin. However, when treated with both ampicillin and SIM, the double mutant became highly sensitive, mirroring the synthetic lethality seen in MRSA (Fig. 7c). This effect was accompanied by a marked reduction in the PG precursor UDP-M5, reinforcing the idea that SIM disrupts cell-wall synthesis in mevalonate-dependent organisms (Fig. 7d, e).

Together, these findings reveal a conserved vulnerability across Gram-positive pathogens: the intersection of c-di-AMP dysregulation and lipid carrier stress creates a therapeutic window that can be exploited by statins and other C_55_-P-targeting compounds. This opens promising avenues for broad-spectrum adjuvant strategies to combat β-lactam resistance beyond *S. aureus*.

## Discussion

MRSA remains a formidable clinical challenge, as adaptation to β‑lactam pressure has rendered the principal treatment for *S. aureus* ineffective. Our data indicate that statins—especially SIM—exploit a previously underappreciated weakness at the intersection of c‑di‑AMP signaling and lipid carrier stress, restoring β‑lactam sensitivity while imposing a fitness cost on compensatory mutants in host‑like environments. Adaptation under FMM inhibition is statin‑dependent: with ZA, evolution reliably converges on loss‑of‑function in *gdpP* (elevated c‑di‑AMP), whereas SIM uniquely disrupts C_55_‑P synthesis, depletes PG precursors, and weakens the wall—counterselecting *gdpP* mutants because they are especially vulnerable under these conditions.

**Fig. 8:**
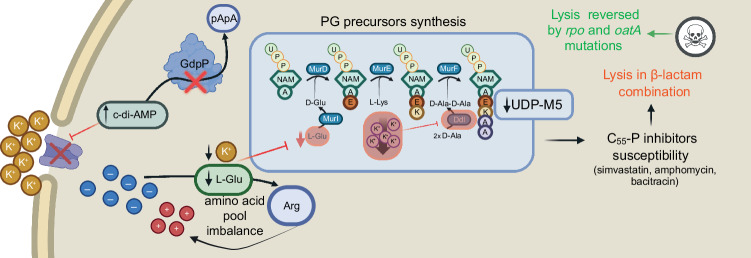
Proposed mechanistic model connecting GdpP, c-di-AMP, and C_55_-P inhibitor sensitivity in *S. aureus.* Inactivation of phosphodiesterase GdpP leads to elevated c-di-AMP levels, which downregulate potassium import systems such as Ktr-like and Kdp-like transporters, resulting in reduced intracellular potassium concentrations. The excess of negative charges from cellular DNA drives the consumption of intracellular glutamate reserves to produce arginine as a compensatory cation. This metabolic shift decreases the availability of glutamate-derived precursors required for peptidoglycan (PG). The reduction of key PG intermediates, such as UDP-MurNAc-pentapeptide (UDP-M5), increases the bacterium’s susceptibility to undecaprenyl phosphate (C_55_-P) inhibitors, leading to inevitable cell lysis when combined with oxacillin. However, this vulnerability can be partially reversed by compensatory *rpo* and *oatA* mutations, albeit at the cost of reduced virulence. Created in BioRender: https://BioRender.com/ut31xih.

Mechanistically, we propose a double bottleneck centered on soluble PG precursors. First, prior work shows that elevated c-di-AMP in *gdpP* mutants impairs K⁺ import^28,39–41^, which can diminish Ddl activity^29^ and lead to UDP-M3 accumulation—a model consistent with our observations. Second, based on the literature, K⁺ limitation perturbs the glutamate–glutamine pool^42–44^; we hypothesize that this may favor diversion of glutamate toward arginine biosynthesis as a compensatory positively charged osmolyte^45^, which would compromise MurD-dependent UDP-M2 synthesis^46^ and further limit PG precursor availability. In line with this model, we observed significantly lower L-glutamate in *gdpP* lysates versus WT (Figs. 4c, 8 and Supplementary Fig. 7b).

This synthesis deficit is accompanied by suppressor mutations in *oatA* (MurNAc O‑acetyltransferase). MurNAc O‑acetylation adjusts to defects in cell-wall biogenesis, partly by tuning autolysin activity^34,36^. We propose that *oatA* suppressors re‑balance PG homeostasis—coordinating synthesis with turnover^30,47^. Consistently, *gdpP* mutants carrying *oatA* suppressors show higher PG content per cell mass, indicating reduced autolytic turnover. Complementing this stabilization, recent work shows *gdpP* mutations often co‑occur with changes that increase PBP4‑mediated crosslinking^48^, suggesting crosslink reinforcement as an alternative way to fortify the wall when GdpP is inactive. A key open question is whether PBP4 activation can offset SIM‑mediated OXA sensitization in our system, tipping the balance back toward resistance despite lipid carrier stress.

How *gdpP* mutations enhance OXA resistance likely involves stringent‑response signaling. Amino‑acid shortages—such as reduced glutamate and glutamine—can elevate (p)ppGpp^23^, promoting antibiotic tolerance^49^. In line with this, we frequently detected mutations in RNA polymerase subunits *rpoB/rpoC* in OXA‑resistant isolates, with or without *gdpP* mutations, echoing prior links between the stringent response and increased MRSA resistance^30,50^. Notably, *rpoB/C* and *gdpP* seem to act through distinct routes, as they exert opposing effects on SIM sensitivity, underscoring divergent resistance strategies within the same adaptive landscape.

Functionally, SIM restores β‑lactam sensitivity in *gdpP* mutants and suppresses infection in vivo, highlighting its therapeutic potential. Crucially, SIM’s efficacy does not hinge on preventing suppressors; rather, any escape from SIM carries a fitness cost. This aligns broad observations across pathogens, where resistance or tolerance mutations frequently burden metabolism and cell-envelope homeostasis^51,52^. By targeting lipid carrier biosynthesis, SIM forces β-lactam-resistant strains into an evolutionary corner in which evasion requires sacrificing virulence. In effect, SIM redirects MRSA’s adaptive landscape toward self-limiting outcomes, creating a therapeutic pressure that is both potent and evolutionarily robust.

Beyond *S. aureus*, the OXA-dependent synthetic lethality between *gdpP* dysfunction and SIM suggests a broader vulnerability shared among Gram-positive pathogens. In *S. pneumoniae*, loss of c-di-AMP phosphodiesterase activity sensitized cells to SIM and β-lactam treatment by lowering UDP-M5, underscoring that SIM’s impact on C_55_-P availability disrupts fundamental cell-wall biosynthesis. This vulnerability is not unique to SIM: other C_55_-P-targeting agents (e.g., amphomycin) also restored β-lactam sensitivity in *gdpP* mutants, indicating that the antagonism stems from interference with lipid carrier recycling rather than the mevalonate pathway alone. Hence, exploiting lipid carrier stress offers a route to resensitize recalcitrant infections while limiting survival and transmission. Systematic characterization of clinical isolates showing SIM–β-lactam antagonism should uncover additional genetic determinants—potentially intersecting with *gdpP*-linked pathways—that prime bacteria for combination therapy. Identifying these nodes will enable adjuvant design that leverages resistance–virulence trade-offs, moving the field toward evolution-informed treatments in which β-lactams are revived and resistance is costly by design.

## Methods

### Ethics statement

All research was conducted in compliance with relevant ethical regulations. Animal experiments were approved by the Regional Government of Madrid under licence PROEX 049.3-24.

### Bacterial strains

The bacterial strains utilized in this study are detailed in Supplementary Table 1. Unless specified otherwise, experiments were conducted using methicillin-resistant *Staphylococcus aureus* (MRSA) strains USA300^53^ and *S. aureus* JE2^54^; *S. pneumoniae* strains used in this study were derived from D39^55^. For cloning purposes, *Escherichia coli* DH5α (Sigma) was employed, while *E. coli* IM08 served as the recipient strain^56^.

### Bacterial growth conditions

*S. aureus* strains were cultured in TSB medium at 37 °C, supplemented where appropriate with erythromycin (2 µg/mL). For infection experiments, wild-type (WT) and mutant *S. aureus* strains were grown overnight at 37 °C with shaking, diluted 1:100 in fresh TSB, and incubated at 37 °C with shaking until reaching an optical density at 600 nm (OD_600_) of 0.6 (exponential phase). Slow-growing mutants were incubated for additional 15–30 min intervals to ensure that all cultures reached the specified OD_600_ range. *E. coli* strains were cultured in Luria-Bertani broth (LB) medium at 37 °C with ampicillin (100 µg/mL) when required. *S. pneumoniae* cells were grown in Todd Hewitt Broth (THB) containing 0.5% (w/v) yeast extract at 37 °C in 5% CO_2_. To avoid precipitation in an aqueous solution, Zaragozic acid (ZA) and Simvastatin (SIM) were prepared in dimethylsulfoxide (DMSO) stock solution and diluted 1:1 in methanol before further dilution in phosphate-buffered saline (PBS) or in *S. aureus* cultures to working concentrations.

### Construction of knock-out mutants

Mutants were generated using a two-step recombination process^57^. Approximately 700 bp upstream and downstream fragments flanking the target gene were amplified and joined via long flanking homology PCR. The resulting fragments were assembled into the pMAD vector using Inverse Touchdown Assembly (ITA) and propagated in *E. coli* IM08. The purified plasmids were then used to transform *S. aureus* via electroporation (1.8 kV, 200 Ω, 25 µF; time constant 4.5–5.0 ms). Following successful integration of the entire plasmid into the genome, strains were grown at 42 °C, plated on TSB X-Gal (50 μg/mL), and white colonies were screened for loss of the plasmid and presence of the desired construct by colony PCR. All resulting constructs were verified by DNA sequencing. Plasmids and primers used in these procedures are listed in Supplementary Table 2 and Supplementary Data 2. Newly generated strains and plasmids are available upon request.

### Long-term evolution experiments

This experiment involved five distinct bacterial backgrounds of *S. aureus* USA300 (LAC), each cultured in TSB supplemented with 0.1× the MIC of OXA. Where indicated, statins were added at a final concentration of 25 µM. Cultures were incubated at 37 °C with shaking (200 rpm) in a final volume of 100 µL, and 1 µL of culture was transferred daily into fresh medium containing the same sub-MIC concentration of OXA (and statins when applicable). This daily passaging was continued for 14 consecutive days, corresponding to approximately 100 bacterial generations. After the initial 14-day passaging period, the OXA MIC was determined using the standard broth microdilution method. Isolates exhibiting increased MIC values were selected for whole-genome sequencing (WGS). Following initial sequencing, selected isolates were subjected to an additional ~100 generations of passaging under the same sub-MIC conditions. Daily transfers of 1 µL into 100 µL of fresh medium were continued, and isolates were re-sequenced at the end of the extended passaging period to capture further genomic evolution.

### Antibiotic resistance determination

Resistance to various antibiotics was determined on agar plates using MIC Test Strips (Liofilchem). Exponential cultures were adjusted to OD_600_ 0.1 in PBS and spread evenly across a TSB agar plate three times using a sterile cotton swab, rotating the plate 60° between each spreading. Strips were applied once the plates had fully dried, and plates were incubated at 37 °C for 16–18 h under aerobic conditions. Alternatively, MICs were determined by a standard microdilution assay. Briefly, exponential cultures were diluted in TSB broth to ~5 × 10^5^ colony-forming units per milliliter (CFU/mL), and serial dilutions of antibiotics were added to 96-well plates. After incubation at 37 °C for 16–20 h, the MIC was recorded as the lowest antibiotic concentration that completely inhibited visible growth.

### Disk diffusion test

A pure bacterial colony from a fresh 18–24 h culture was selected and emulsified in sterile saline (0.85% NaCl) to achieve a turbidity equivalent to OD_600_ 0.1. The bacterial suspension was then evenly spread onto the surface of TSB agar plates using a sterile cotton swab. The swab was passed over the agar surface in three different directions to ensure even distribution of the inoculum, and the plate was allowed to dry for 3–5 min. Antibiotic-impregnated disks were applied to the inoculated agar surface using sterile forceps. The antibiotic disks were placed at least 24 mm apart to avoid overlapping zones of inhibition. Once the disks were positioned, the plates were inverted and incubated at 37 °C for 16–18 h under aerobic conditions.

### Checkerboard assay and isobologram construction

The interaction between SIM and OXA was evaluated using a two-dimensional checkerboard microdilution assay with two-fold serial dilutions of OXA and SIM. Assays were performed in biological triplicate. For each replicate, the MIC was defined as the lowest concentration that prevented visible growth, and FIC values were calculated independently. Reported MIC and FIC values correspond to the median values across biological replicates. For each assay, the MICs of OXA and SIM alone and in combination were used to calculate the fractional inhibitory concentration (FIC):

FIC_OXA_ = MIC_OXA_ in combination/MIC_OXA_ alone

FIC_SIM_ = MIC_SIM_ in combination/MIC_SIM_ alone.

The FIC index was calculated as: FIC index = FIC_OXA_ + FIC_SIM._ Interactions were classified as synergistic when the FIC index was ≤ 0.5, additive or non-synergistic when >0.5 to ≤1.0, indifferent when >1.0 to ≤4.0, and antagonistic when >4.0. Reported FIC values correspond to the representative inhibitory combination shown in the checkerboard and were consistent across biological replicates.

Normalized isobolograms were generated in R using *ggplot2* to visualize the interaction between SIM and OXA. The x-axis represents the fractional OXA concentration, calculated as the concentration of OXA in combination divided by the MIC of OXA alone. The y-axis represents the fractional SIM concentration, calculated as the concentration of SIM in combination divided by the MIC of SIM alone. Thus, the single-agent MICs were normalized to (1, 0) for OXA alone and (0, 1) for SIM alone. The selected inhibitory combination was plotted according to its FIC values. The dashed diagonal line connecting (0, 1) and (1, 0) represents the theoretical line of additivity. A smooth curved isobole was drawn through the single-agent MIC points and the selected combination point using quadratic Bézier interpolation for visualization only. FIC index values and interaction classifications were calculated directly from the experimental MIC values and were not affected by the plotted curve.

### Development of protein overexpression strains

The pAmy plasmids were used for protein overexpression or complementation in *S. aureus*^26^. Coding sequences were amplified and, when applicable, assembled into the pAmy vector using Inverse Touchdown Assembly (ITA). All plasmids were sequence-verified and integrated chromosomally at the *amyE* neutral locus of *S. aureus*^26,57^. Constructs were introduced into *S. aureus* by electroporation (1.8 kV, 200 Ω, 25 µF; time constant 4.5–5.0 ms). Correct integration and orientation of the inserts were confirmed by PCR using primers annealing to the *amy* locus and/or the inserted coding sequence.

### Determination of bacterial survival

Bacterial survival following treatments was primarily assessed by quantifying CFU/mL. Treated cultures were serially diluted in sterile PBS to obtain appropriate ranges, and 100 µL of each dilution was spread in triplicate onto agar plates. Plates were incubated under standard growth conditions: TSB agar at 37 °C overnight for *S. aureus* and THB agar with 5% horse blood at 37 °C with 5% CO₂ for 20–24 h for *S. pneumoniae*. Colonies on plates containing 30–300 colonies were counted, and CFU/mL was calculated by multiplying the number of colonies by the dilution factor and dividing by the plated volume. Percentage survival was determined by dividing the CFU/mL of treated samples by that of untreated controls and multiplying by 100. Alternatively, a spot assay was used to evaluate survival: treated cultures were serially diluted in PBS, and 5–10 µL of each dilution was spotted onto appropriate agar plates. Plates were allowed to absorb the inoculum and incubated under the same conditions until colonies were visible.

### Whole-genome sequencing

Genomic DNA was extracted using the GeneJET Genomic DNA Purification Kit and quantified with the Qubit dsDNA HS Assay Kit (Thermo Scientific). 1 ng of each DNA sample was used to generate the genomic libraries following the manufacturer’s recommendations (Nextera XT DNA Sample Preparation Kit, Illumina). DNA libraries were then pooled in equimolar proportions and sequenced employing a MiSeq Reagent Kit V2 (Illumina). Paired-end 2 × 300 bp reads were generated on an Illumina MiSeq instrument. Sequences of the isogenic WT strain (USA300_FPR3757) were determined in parallel.

The sequences were analyzed using the open, web-based computational platform Galaxy (https://usegalaxy.org/)^58^. Mapping trimmed reads to the reference USA300 FPR3757 genome (Taxonomy ID: 451515) was performed by the BWA-MEM^59^ algorithm. After read alignment, Picard tools were used for the parent strains and each suppressor to mark and remove duplicate sequences mapping to different regions. Subsequently, SNPs (single-nucleotide polymorphisms) and indels (insertions and deletions) were detected by comparison of the results obtained after detecting genetic variants by FreeBayes^60^ and VarScan^61,62^.

### Gene expression analysis by quantitative reverse-transcription PCR (qRT-PCR)

*S. aureus* cultures were harvested, and the cells were resuspended in lysis buffer (20 mM Tris-HCl pH 8, 10 mM EDTA) supplemented with lysostaphin (0.1 mg/mL) and incubated at 37 °C for 15 min. Total RNA was isolated using the RNeasy kit (Qiagen). RNA samples were treated with DNase I (New England Biolabs) to remove all DNA traces. RNA purity and quality were determined using Nanodrop technology (Thermo Scientific). DNA-free RNA samples were used for cDNA synthesis, employing Superscript III reverse transcriptase (Applied Biosystems) and random hexamer primers. Each qRT-PCR was run in triplicate using the Universal SYBR Green Master mix (Applied Biosystems). The designed primers were used to amplify a 120–200 bp DNA fragment. Relative amplification was calculated using the 2^–∆∆CT^ Livak Method^63^. The housekeeping gene *gyrB* was used as a reference gene for normalization.

### Fitness and growth measurements

To assess the fitness of *S. aureus*, overnight cultures were diluted 10-fold in TSB, and OD_600_ was measured using an Eon plate reader (Biotek, USA). For continuous growth monitoring, at least three replicates per strain and condition tested were grown in three independent experiments in 200 μL medium in a 96-well plate inoculated 1:1000 from exponentially growing precultures. OD_600_ was monitored in the plate reader at 10 min intervals, using the optimal growth temperature for the tested species.

### Microscopy

Bacteria were immobilized on LB pads containing 1% (w/v) agarose. Phase contrast microscopy was performed using a Zeiss Axio Imager Z2 microscope (Zeiss, Germany) equipped with a Plan-Apochromat 63X phase contrast objective lens and an ORCA-Flash 4.0 LT digital CMOS camera (Hamamatsu Photonics, Japan), using the Zeiss Zen Blue [v2.0.0.0] software. Image analysis and processing were performed using the Fiji/ImageJ version 1.53 software^64^. The MicrobeJ plugin was used for cell width and length measurements, using manually edited cell outlines as needed^65^. Microscopy images shown are representative of three biological replicates.

### Staphyloxanthin measurement

Staphyloxanthin production was assessed using a modified protocol^66^. *S. aureus* cultures were normalized by CFU/mL. After harvesting, pellets were resuspended in 10 mL of methanol and incubated for 30 min at 60 °C with continuous agitation. The samples were then centrifuged to remove cell debris, and the pigment-containing supernatant was concentrated using a *SpeedVac*. The resulting pellet was vortexed in 20 µL of PBS containing glass beads, followed by the addition of 180 µL of methanol. The samples were then shaken at 1000 rpm at 55 °C for 4 min. After centrifugation at maximum speed for 10 min, the absorbance of the supernatant was measured at OD_465_.

### Quantification of C_55_-OH and C_55_-P in bacterial membrane lipid extracts

#### Preparation of *S. aureus* membranes

*S. aureus* cells were grown overnight at 37 °C in 10 mL of TSB or LB (pH 7), respectively. The cells were washed, collected by centrifugation, and resuspended in TSB. A 1:200 dilution of this suspension was inoculated into 200 mL of the experimental medium. Once the culture reached an OD_600_ of 0.6 and normalized, cells were harvested by centrifugation, resuspended in 10 mL of PBS, and lysed using a French press. Cell debris was removed by centrifugation, and the supernatant was subjected to ultracentrifugation at 270,000 × *g* for 15 min using a Beckman Optima Max TL ultracentrifuge. Membrane pellets were collected for lipid extraction as described below.

#### Extraction of lipids from membrane fractions

Membrane lipids from *S. aureus* were extracted following a modified protocol^22,67^. Membrane pellets were resuspended in 500 µL of PBS, followed by the addition of 1.25 mL of methanol and 625 µL of chloroform. The mixture was vortexed for 2 min at room temperature and centrifuged at 7100 × *g* for 10 min at 4 °C. The supernatant was collected, and 625 µL each of chloroform and PBS were added. After vortexing and a second centrifugation at 7100 × *g* for 10 min at 4 °C, the chloroform phase was isolated and vacuum-dried. The dried lipid pellets were resuspended in 300 µL of UPLC mobile phase solvents (50% H_2_O with 0.1% (v/v) formic acid + 50% isopropanol with 0.1% (v/v) formic acid). To validate the extraction method for C_55_-OH and C_55_-P, 200 nmol of each lipid standard (Larodan) was added to fresh purified membrane samples before extraction.

#### Detection of C_55_-OH and C_55_-P by UPLC

Lipid extracts resuspended in UPLC mobile phase solvents were analyzed using UPLC (Acquity H-Class, Waters) on a reverse-phase C18 column (Kinetex C18, 1.7 µm particle size, 100 Å pore size, 50 × 2.1 mm, Phenomenex). Lipid separation was performed with a gradient of H_2_O containing 0.1% (v/v) formic acid (solvent A) and isopropanol with 0.1% (v/v) formic acid (solvent B), at a flow rate of 0.4 mL/min over 8 min. The column temperature was maintained at 60 °C, and detection was carried out at a wavelength of 210 nm. C_55_-OH and C_55_-P were identified based on their retention times compared to standards, and their abundances were calculated relative to the total peak area of each chromatogram. For mutant-to-WT ratio calculations, samples from independent cultures grown on the same day were randomly paired. UPLC data collection and analysis were performed using Empower 3 chromatography software (Waters).

### Isoprenoid diphosphates extraction

FPP was extracted using a modified method previously described by Vallon *et al*^68^. and Krute *et al*^69^. An FPP standard (Sigma) was prepared in acetonitrile. Samples were dried with *SpeedVac* and extracted with 1 mL chloroform. Particulate matter was separated and dried again with *SpeedVac*. Samples were redissolved in 100 µL of methanol + 10 mM ammonium hydroxide (7:3). The samples were filtered for further analysis in a UPLC as described above. FPP separation was performed with a gradient of H_2_O containing 0.1% (v/v) formic acid (solvent A) and isopropanol with 0.1% (v/v) formic acid (solvent B), at a flow rate of 0.4 mL/min over 8 min. The column temperature was maintained at 60 °C, and detection was carried out at a wavelength of 210 nm. FPP was identified based on its retention times compared to standards, and its abundances were calculated relative to the total peak area of each chromatogram. For mutant-to-WT ratio calculations, samples from independent cultures grown on the same day were randomly paired. UPLC data collection and analysis were performed using Empower 3 chromatography software (Waters).

### Triton X-100 survival assay

*S. aureus* cultures were harvested by centrifugation, washed once with 1 mL ice-cold H₂O, and resuspended in 1 mL of 50 mM Tris-HCl (pH 7.5) containing 0.05% (v/v) Triton X-100. Samples were incubated at 37 °C for 4 h. Viable counts were determined at the start of the assay (0 h) and after 4 h by serial dilution and plating. Survival was calculated as:

% survival = CFU/mL at 4 h ÷ CFU/mL at 0 h × 100

All measurements were performed using independently prepared biological replicates.

### PG isolation

An established procedure was adopted^70^ albeit with significant changes, to purify the PG of the strains under investigation. In summary, 200 mL cultures were grown to an OD_600_ of 0.5, and cells were harvested at 3000 × *g* for 15 min. Pellets were boiled with stirring with SDS 5% (w/v) for 3 h. Lysates were stirred overnight. Pellets were further washed by ultracentrifugation for 10 min at 20 °C and 150,000 × *g* three times.

For *S. aureus* PG isolation, glass beads with a diameter of 0.1 mm and a Mini-Beadbeater were used to mechanically lyse the cell pellets. After ultracentrifuging the supernatants, the pellets were resuspended in 1 mL Tris-HCl 100 mM pH 7.5 and subjected to 2 h of treatment with 40 µL MgSO_4_ 1 M, 2 µL RNase A (500 µg/mL), and 1 µL DNase I (100 µg/mL) at 37 °C, followed by an overnight treatment with 50 µL CaCl_2_ and 100 µL trypsin (2 mg/mL) at 37 °C. Samples were centrifuged at 20,000 × *g*, and the pellet was treated with 1 mL LiCl 8 M, 1 mL EDTA (ethylene diamine tetra-acetic acid) 100 mM, and 1 mL of acetone. Teichoic acids were eliminated from the pellets by resuspension in 1 mL of 48% hydrofluoric acid (v/v) and shaking incubation for 48 h at 4 °C. After four rounds of washing with cold Milli-Q water, the pellets containing PG sacculi were obtained. Pellets containing purified PG sacculi were finally resuspended in water, and muramidase was added to the reaction at a final concentration of 100 µg/mL. The reactions were incubated at 37 °C overnight and stopped by boiling the reaction for 5 min. The supernatants (soluble muropeptides) were subjected to sample reduction. First, pH was adjusted to 8.5-9 by addition of borate buffer 0.5 M pH 9, and then muramic acid residues were reduced by sodium borohydride treatment (NaBH_4_ 10 mg/mL final concentration) for 30 min at room temperature. Finally, pH was adjusted to 2.0–4.0 with orthophosphoric acid 25% (v/v) prior to analysis by LC-MS.

### PG amount quantification

Following purification, PG sacculi were collected by centrifugation and resuspended in ultrapure water. Suspensions were transferred to pre-weighed microcentrifuge tubes and freeze-dried until complete removal of moisture. After lyophilization, samples were equilibrated to room temperature in a desiccator, and PG dry mass was determined by subtracting the pre-recorded tube weight from the final tube weight.

For PG quantification, cultures were harvested in mid-log phase at OD_600_ ≈ 0.5, and PG sacculi were purified from defined culture volumes. In parallel, matched aliquots from the same cultures were serially diluted and plated to determine viable cell counts as CFU/mL. These CFU values were used to account for differences in viable cell number between strains at equivalent OD_600_. For comparisons between different genetic backgrounds, PG amounts were corrected to WT-equivalent viable cell number. For each biological replicate, the PG amount measured for each mutant strain was normalized to the viable cell number of the paired WT culture using:

CFU-corrected PG amount = raw PG amount × paired WT CFU/mL ÷ sample CFU/mL

WT samples were assigned a correction factor of 1. For comparisons within the same strain across treatment conditions, PG amounts were normalized to the harvested culture input using the corresponding OD_600_ and CFU/mL values, without applying a paired WT correction. PG amounts were expressed as milligrams per liter of culture and, where indicated, as CFU-corrected values. All measurements were performed using independently prepared biological replicates, and results are reported as mean ± standard deviation. Raw PG amounts, OD_600_ values, CFU/mL counts, correction factors, and final corrected values are provided in the source data.

### Quantification of intracellular L-glutamate via FDAA derivatization

Intracellular L-glutamate (L-Glu) was quantified by derivatization with Marfey’s reagent (FDAA; 1-fluoro-2,4-dinitrophenyl-5-L-alanine amide). Cell extracts (100 µL) were placed in 1.0 mL Thermo Scientific™ Reacti-Vial™ Small Reaction Vial (Product No. TS-13221 clear; TS-13097 amber). To each vial, 200 µL of 1% FDAA in acetone and 40 µL of 1.0 M sodium bicarbonate (NaHCO_3_) were added to provide alkaline conditions. Reactions were incubated at 40 °C for 1 h. After cooling to room temperature, reactions were quenched with 20 µL of 2 M HCl. Samples were degassed and analyzed directly by LC-MS.

Derivatized L-Glu was detected based on the acetone (C_3_H_6_O) adduct (*m/z* 458.15 [M + C_3_H_6_O + H]^+^). A derivatized L-Glu standard (1 mM) was used as a control for quantification.

### Intracellular soluble muropeptide analysis

To determine the presence and levels of intracellular soluble muropeptides, bacteria were grown until late exponential phase (roughly OD_600_ 0.7) in TSB media before being cooled on ice for 10 min and normalized to the same CFU/mL before extraction, using the strain-specific OD_600_-to-CFU/mL relationship determined from growth-matched cultures. Cells were then harvested by centrifugation at 10,000 × *g* for 10 min. The supernatants were discarded, and the cell pellets were washed three times in ice-cold 0.9% (w/v) NaCl, resuspended in 0.9% (w/v) NaCl so that the cells are 20 times concentrated, and boiled for 10 min before centrifugation at maximum speed in a benchtop centrifuge for 10 min to remove the proteins and insoluble fraction. The supernatants were further analyzed by LC-MS.

### LC-MS analysis

Chromatographic analyses of muropeptides were performed by ultra-performance liquid chromatography (UPLC) on an Acquity H-Class UPLC system (Waters) equipped with a trapping cartridge precolumn (SecurityGuard ULTRA Cartridge UHPLC C18 2.1 mm, Phenomenex) and a reverse-phase C18 column (Kinetex C18, 1.7 µm particle size, 100 Å pore size, 50 × 2.1 mm, Phenomenex) maintained at 45 °C. Muropeptides were detected by measuring the absorbance at 204 nm using an ACQUITY UPLC-UV−visible Detector. Muropeptides were separated using a linear gradient from buffer A (Water + 0.1% (v/v) formic acid) to buffer B (Acetonitrile + 0.1% (v/v) formic acid) over 15 min with a flow rate of 0.25 mL/min. The QTOF instrument was operated in positive ion mode, with data collection performed in untargeted MSe mode. The parameters were set as follows: capillary voltage 3.0 kV, source temperature 120 °C, desolvation temperature 350 °C, sample cone voltage 40 V, cone gas flow 100 L/h, and desolvation gas flow 500 L/h. Mass spectra were acquired at a speed of 0.25 s/scan. The scan was in a range of 100–2000 *m/z*. Data acquisition and processing were performed using MassLynx or UNIFI software package (Waters Corp.). The quantification of muropeptides was based on their relative abundances (relative area of the corresponding peak) and relative molar abundances. A table of all the identified muropeptides and the observed ions is provided (Supplementary Table 3).

### Lipid II extraction and LC-MS analysis of delipidated Gly_5_-Lipid II

Gly_5_-Lipid II was extracted as previously described in ref. ^71^, with some modifications. Two-liter cultures of *S. aureus* strains were grown at 37 °C with shaking to an OD_600_ of 0.7, normalized to the same CFU/mL before extraction, using the strain-specific OD_600_-to-CFU/mL relationship determined from growth-matched cultures, and harvested by centrifugation. The pellets were resuspended in 15 mL PBS (pH 7.4), 52.5 mL CHCl_3_:MeOH (1:2) was added, and the mixture was stirred for 1 h at RT to allow cell lysis. The mixture was poured into two Teflon tubes and centrifuged at 4000 × *g* for 10 min at 4 °C. The supernatant containing the solubilized cellular contents was collected. The supernatants of two tubes were combined and poured into a flask containing 30 mL CHCl_3_ and 22.5 mL PBS (pH 7.4). For 1 h, the liquid was swirled rapidly to combine the layers thoroughly. The homogenized mixture was poured into three clean Teflon tubes and centrifuged at 4000 × *g* for 10 min at 4 °C. An interface fraction was revealed in each Teflon tube between the top aqueous and bottom organic layers. The aqueous layer was progressively removed to get to the interface fraction. The combined interface was dried on a *SpeedVac*. In the second extraction (to remove UDP-M5), the combined dried interface was dissolved in a 15 mL organic mixture of 6 M pyridinium acetate: n-butanol (1:2) (6 M pyridinium acetate was prepared by mixing 51.5 mL glacial acetic acid with 48.5 mL pyridine), and washed with 15 mL of aqueous solvent (n-butanol saturated water) in a separatory funnel. The aqueous layer was extracted again with 10 mL organic solvent (1:2, 6 M pyridinium acetate: n-butanol) to maximize Lipid II extraction. The organic layers were combined and washed with aqueous solvent (n-butanol saturated water) three times (10 mL × 3) to remove the water-soluble UDP-M5. On *SpeedVac*, the pure organic layer was concentrated before being redissolved in DMSO. To remove the Lipid II tail, the sample resuspended in DMSO was incubated with 800 µL of water and 100 µL of 0.1 M ammonium acetate pH 4.2. The mixture was boiled at 100 °C for 90 min, dried on *SpeedVac*, resuspended in 50 µL of water, and centrifuged at 16,000 × *g* for 10 min to remove the precipitate. The pH of the supernatant was adjusted to pH 3-4, and the sample was subjected to LC-MS analysis as described above

### Phylogenetic analysis of GdpP and MvaA orthologs

To investigate the distribution of GdpP and MvaA across diverse bacterial species, orthologous sequences were identified using OrthoDB (version 11)^72^, a comprehensive catalog of orthologs across the tree of life. Following identification (246344at91061 for c-di-AMP phosphodiesterase GdpP and 340903at91061 for hydroxymethylglutaryl-CoA reductase MvaA), a phylogenetic tree representing all species present in OrthoDB was generated using PhyloT v2 (Biobyte), which produces taxonomically accurate trees based on NCBI taxonomy. The identified GdpP and MvaA orthologs were subsequently mapped onto this phylogenetic framework using iTOL (Interactive Tree of Life)^73^, allowing visualization of the evolutionary relationships and distribution patterns of these proteins across bacterial lineages

### Protein structure prediction and visualization

The predicted structure of the *Staphylococcus aureus* GdpP protein was obtained using AlphaFold^74^. The GdpP amino acid sequence (UniProt accession A0A6B0CXK2) was retrieved from UniProt, and the corresponding AlphaFold-predicted model was obtained from the AlphaFold Protein Structure Database^75^. Structural visualization and figure rendering were performed using UCSF ChimeraX (version 1.10.1)^76^.

### Mouse infection pneumonia model

BALB/c OlaHsd *Mus musculus* mice (8 weeks old, body weight 16–19 g) were purchased from Envigo and housed in polypropylene cages under standardized lighting conditions, with *ad libitum* access to food and water. For CFU assays, mice aged 8–10 weeks (*n* = 7 per group) were infected via nasal instillation with 2 × 10^8^ CFU of *S. aureus*. Infection was allowed to progress for 24 h, after which animals were euthanized, and lungs were aseptically collected, homogenized, and plated on TSB agar for CFU enumeration. CFU experiments were performed using both male and female mice. All animal procedures were approved by the Regional Government of Madrid, Spain (license PROEX 049.3-24), and conducted in strict accordance with Spanish legislation and EU Directive 2010/63/EU on animal care and experimentation.

### Differentiation of bone marrow–derived macrophages

Macrophages were differentiated from bone marrow cell suspensions obtained from BALB/c OlaHsd mice. Cells were seeded in non-treated 60‑mm Petri dishes containing high glucose-DMEM (Biowest) supplemented with 10% FBS (Sigma), penicillin-streptomycin solution, 2 mM L-glutamine (Biowest), 1 mM sodium pyruvate (Biowest), and 20 ng/mL M-CSF (PeproTech), at a density of 1 × 10^7^ cells per plate in 5 mL. Cultures were maintained for 7 days at 37 °C and 5% CO₂. Fresh medium was added on day 3. On day 7, macrophages were harvested and counted, yielding cultures composed of approximately 95% macrophages.

### Macrophage survival assays

Bone marrow–derived macrophages were seeded in 24‑well plates 24 h prior to infection at a density of 2 × 10^5^ cells per well in 0.4 mL of DMEM supplemented with 10% FBS, 2 mM L-glutamine, 1 mM sodium pyruvate, and 20 ng/mL M-CSF, without antibiotics. Infections were performed after 24 h at multiplicities of infection (MOI) of 2, 5, 10, and 20 by adding 0.1 mL of bacterial suspensions diluted in complete medium. Plates were centrifuged at 500 × *g* for 5 min at room temperature and incubated at 37 °C in a humidified 5% CO₂ atmosphere. After 90 min of infection, extracellular bacteria were eliminated by replacing the medium with fresh medium containing 10 μg/mL lysostaphin (Ambi Products LLC, LSPN‑50) and 100 μg/mL gentamycin, followed by incubation for 30 min at 37 °C. Cells were washed twice and cultured in supplemented DMEM, without antibiotics, and, when indicated, with 0.25 mg/mL OXA, 0.025 mM SIM, or both. This time was defined as time point 0 h. At 24 h post-infection, macrophages were harvested, and cell survival was determined as the percentage of trypan blue–negative macrophages normalized to the number of viable macrophages at time 0 h.

### Crystal violet biofilm staining assay

Biofilm formation was assessed using a crystal violet (CV) staining assay as previously described^77^. Overnight cultures of *S. aureus* strains were grown without shaking at 37 °C in TSB and diluted 1:200 in 66% TSB supplemented with 0.5% glucose. Two hundred microliters of the diluted cultures were added to wells of 96-well, flat-bottom, tissue culture-treated plates (Corning, Corning, NY) and incubated overnight at 37 °C. Non-adherent cells were removed by gently overturning the plate and washing three times with PBS. Biofilms were fixed with 205 μL of 100% ethanol, allowed to dry, and stained with 205 μL of 0.1% CV for 15 min at room temperature. Excess dye was removed by washing three times with ddH_2_O, and plates were dried overnight. CV was eluted using 205 μL of elution buffer (ddH_2_O containing 40 mM HCl and one-third volume ethanol), and 80 μL of eluate was transferred to a new 96-well plate for quantification at 595 nm.

### Statistics & reproducibility

No statistical method was used to predetermine sample size. Sample sizes were selected based on established experimental practice and are reported in the figure legends. LTEE lineages showing no change in OXA MIC after 200 generations were excluded from further analysis according to the study design; no other data were excluded. The experiments were not randomized. The investigators were not blinded to allocation during experiments and outcome assessment. Statistical analyses were performed using GraphPad Prism 8.0, with the tests, exact *n* values, and replicate definitions reported in the corresponding figure legends. Experiments were performed using independent biological replicates and reproduced with similar results.

### Data analysis and representation

Data were plotted and analyzed using Prism 8.0 (GraphPad Software). Statistical significance was assessed using unpaired two-tailed Student’s *t*-tests (with Welch’s correction when appropriate) or one-way ANOVA, as indicated. A *p*-value of less than 0.05 was considered statistically significant. All assays were performed with at least three independent biological replicates, unless stated otherwise.

### Reporting summary

Further information on research design is available in the Nature Portfolio Reporting Summary linked to this article.

## Supplementary information

Supplementary information

Description of Additional Supplementary File

Supplementary Data 1

Supplementary Data 2

Reporting summary

Transparent Peer Review file

## Source data

Source Data

## Data Availability

The whole-genome sequencing data generated in this study have been deposited in the European Nucleotide Archive under accession code PRJEB108212. The remaining data generated in this study are provided in the Supplementary Information and Source Data file. Source data are provided with this paper.
