# Supplementary material for: Statin-induced lipid carrier stress reveals a conserved vulnerability in β-lactam-resistant Gram-positive bacteria
Source: Nat Commun. 2026 Jul 20;17:6680. doi: 10.1038/s41467-026-75729-8 (PMC13385884; doi:10.1038/s41467-026-75729-8)
Supplement: Supplementary file 1 — Supplementary information [file 41467_2026_75729_MOESM1_ESM.pdf]

SUPPLEMENTARY INFORMATION

Statin-induced lipid carrier stress reveals a conserved vulnerability in β-lactam-resistant Gram-positive bacteria

Gabriel Torrens[1], Sean W. Bisset[1], Maria López-Bravo[2], Anders F. Johansson[3], Daniel Lopez[2] and Felipe Cava[1,4]*

Affiliations:

[1]Department of Molecular Biology, Umeå University, Umeå, SE-901 87, Sweden.

[2]National Centre for Biotechnology, Spanish National Research Council (CNB-CSIC), Madrid, 28049 Spain

[3]Department of Clinical Microbiology, Umeå University, SE-901 85 Umeå, Sweden

[4]The Laboratory for Molecular Infection Medicine Sweden (MIMS). Umeå Center for Microbial Research (UCMR). Science for Life Laboratory (SciLifeLab), Umeå, SE-901 87, Sweden.

***For correspondence:** felipe.cava@umu.se

Running title: Peptidoglycan Carrier Inhibition in Resistant MRSA

Keywords: Methicillin-resistant *Staphylococcus aureus* (MRSA), β-lactam resistance, *gdpP*, cyclic di-AMP signaling, mevalonate pathway, synthetic lethality

Table of contents:

- Supplementary Figures 1-10.
- Supplementary Table 1 - bacterial strains
- Supplementary Table 2 - plasmids
- Supplementary Table 3 - identified muropeptides
- Supplementary References

Supplementary Figures

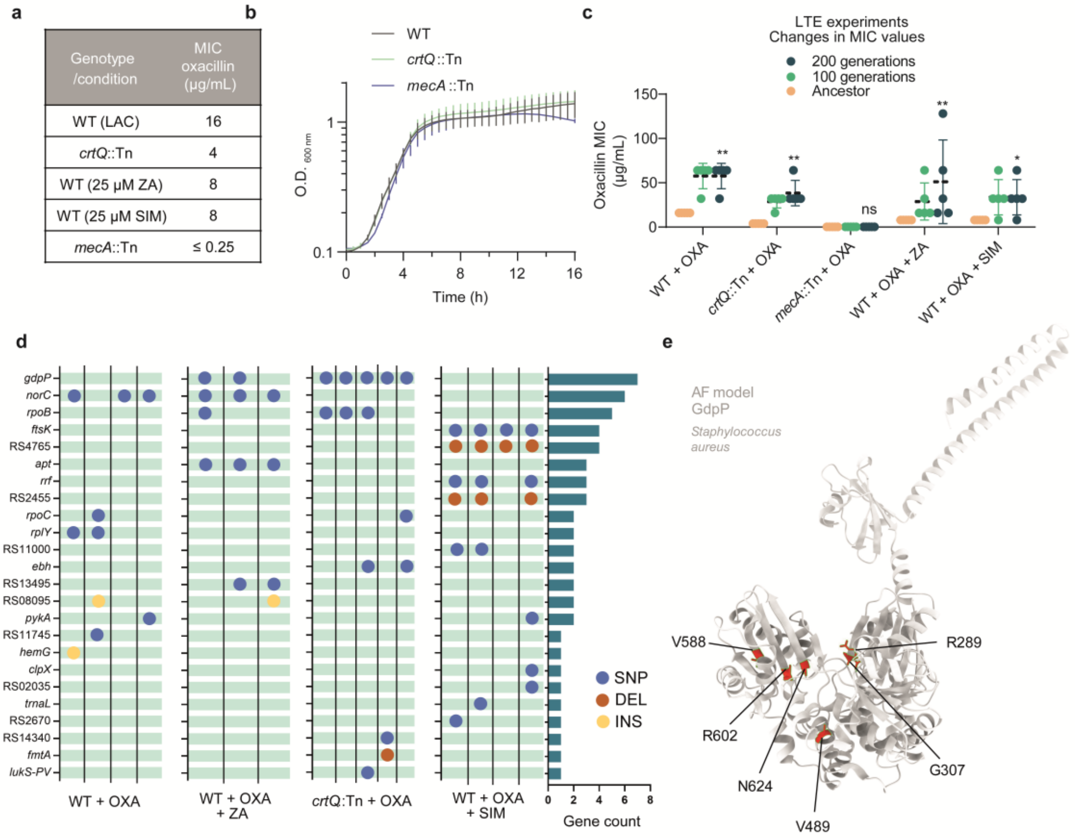

Supplementary Fig. 1. Evolution of oxacillin resistance and GdpP loss-of-function in *S. aureus* under statin exposure. (**a**) Oxacillin minimum inhibitory concentration (MIC) for the strains utilized in the long-term evolution experiment (LTEE). (**b**) Growth curves of WT, and *crtQ::*Tn and *mecA::*Tn transposon mutants. Data are mean ± standard deviation from n = 3 independent biological replicates. (**c**) Changes in oxacillin MIC across successive generations in different lineages for each strain tested in the LTEE experiment. Data are mean ± standard deviation from n = 3 independent biological replicates. Statistical significance was determined using the Mann–Whitney U test (*p < 0.05; **p < 0.01; ***p < 0.001; ****p < 0.0001; ns, not significant). (**d**) Identification of genetic variants in the LTEE, showing genes affected by single nucleotide polymorphisms (SNPs), deletions (DEL) and insertions (INS) after 200 generations of exposure to sub-MIC concentrations of oxacillin with and without ZA and SIM (25 μM). Strain backgrounds: WT and *crtQ::*Tn. (**e**) Predicted structure of GdpP from *S. aureus*

(UniProt: A0A6B0CXK2) generated by AlphaFold and visualized in ChimeraX. SNPs and frameshift mutations associated with loss of function in the LTEE experiment are highlighted in red. OXA, Oxacillin; SIM, Simvastatin; ZA, Zaragozic acid. Source data are provided as a Source Data file.

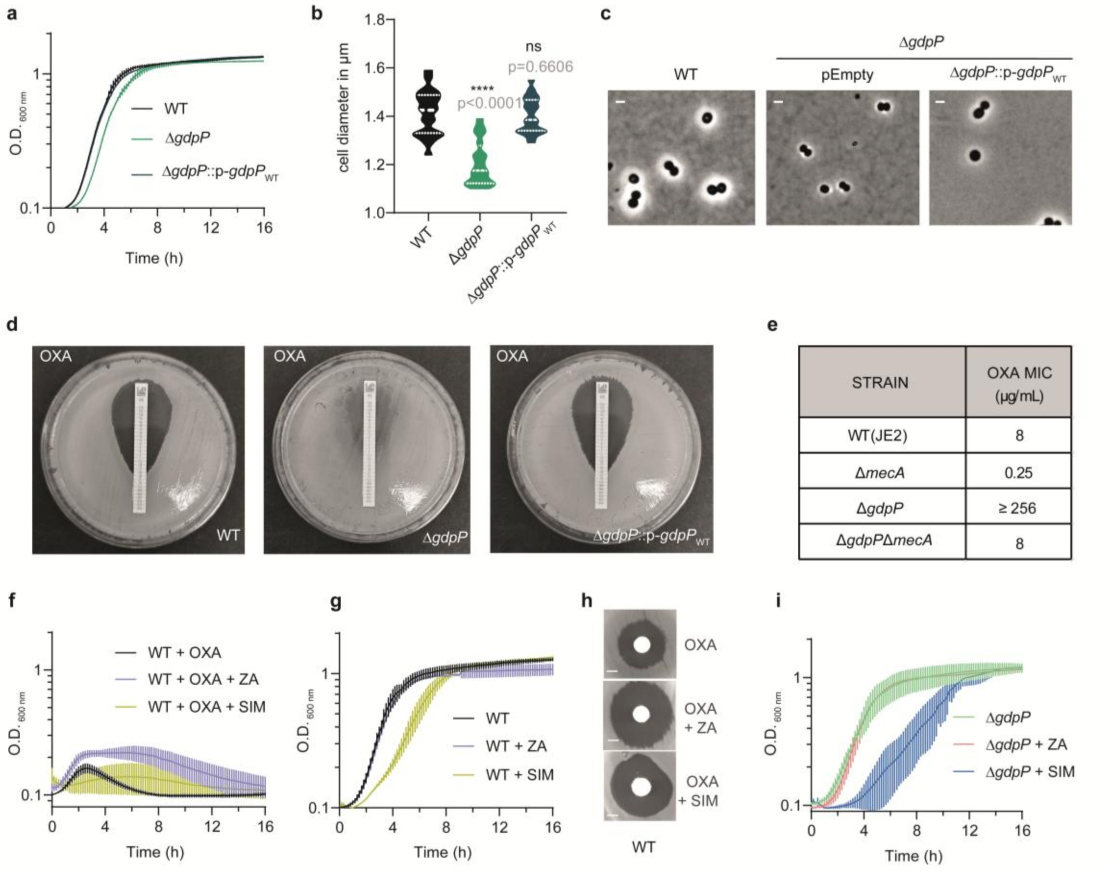

Supplementary Fig. 2. GdpP-mediated oxacillin resistance and cell morphology in *S. aureus* and their modulation by statins. (**a**) Growth curves of the WT strain (JE2), *gdpP* mutant, and complemented *gdpP* mutant. (**b**) Mean cell diameter (*n* = 20 individual cells per strain) and (**c**) representative microscopy images of WT, *gdpP* mutant and complemented strain (scale bar: 1 µm). Statistical significance was assessed using the Student's *t*-test (unpaired, two-tailed) with significance indicated as *$p < 0.05$; **$p < 0.01$; ***$p < 0.001$; ****$p < 0.0001$; ns, not significant. (**d**) Oxacillin resistance of WT, *gdpP* mutant and complemented strain, assayed using Oxacillin E-test® strips. (**e**) Oxacillin minimum inhibitory concentrations (MICs) determined by broth microdilution for WT, *mecA* and *gdpP* single mutants and the double mutant. (**f**) Growth curves of WT strain treated with or without statins at 25 µM in the presence of 16 µg/mL of OXA. (**g**) Growth curves of the WT strain treated with and without statins (25 µM). (**h**) Disk diffusion test of OXA (5 µg/disc) and statins (25 nmol/disc) on TSB for WT strain (scale bar: 10 mm). (**i**) Growth curves of the *gdpP* mutant strain treated with and without statins (25 µM). For

panels a, f, g and i, data are mean ± standard deviation from $n = 3$ independent biological replicates. OXA: Oxacillin; SIM: Simvastatin; ZA: Zaragozic acid. Source data are provided as a Source Data file.

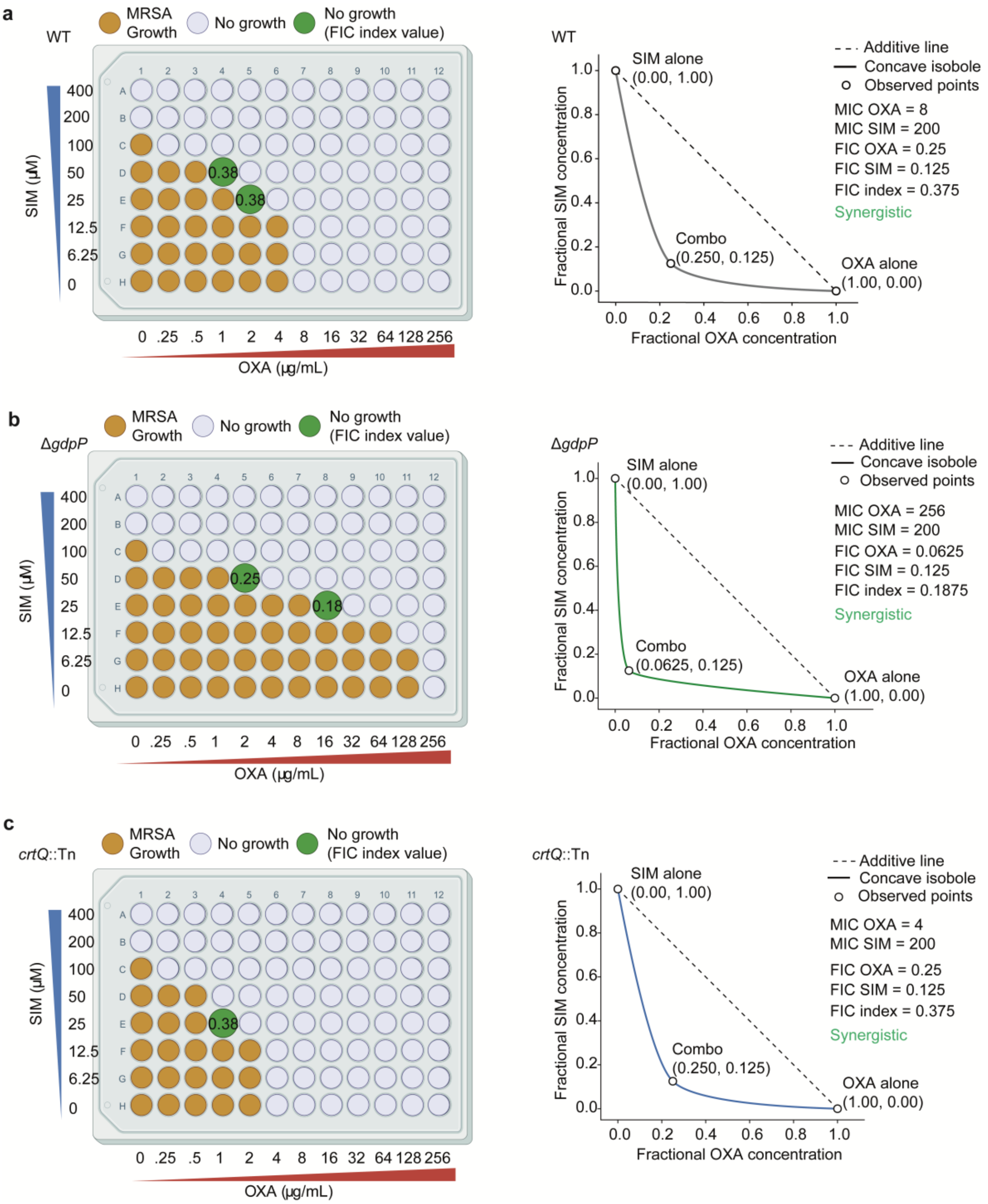

Supplementary Fig. 3. Checkerboard analysis of oxacillin and simvastatin interactions in WT, Δ*gdpP*, and *crtQ*::Tn strains. (a–c) Representative checkerboard assays using two-fold serial dilutions of oxacillin (OXA, 0–256 μg/mL) and simvastatin (SIM, 0–400 μM) are shown on the left, with the corresponding isobologram on the right, for WT (**a**), Δ*gdpP* (**b**), and *crtQ*::Tn (**c**) strains. Orange circles indicate visible growth, light-blue circles indicate no visible growth, and green circles indicate inhibitory

combinations used for fractional inhibitory concentration (FIC) calculation. In the isobolograms, drug concentrations are normalized to the MIC of each compound alone; open circles indicate observed inhibitory points, the dashed line indicates the theoretical additive line, and the solid curve indicates the fitted isobole. FIC index values are shown in each panel. Data are mean ± standard deviation from n = 3 independent biological replicates. Panels a–c were created in BioRender: https://BioRender.com/99mpdfr. Source data are provided as a Source Data file.

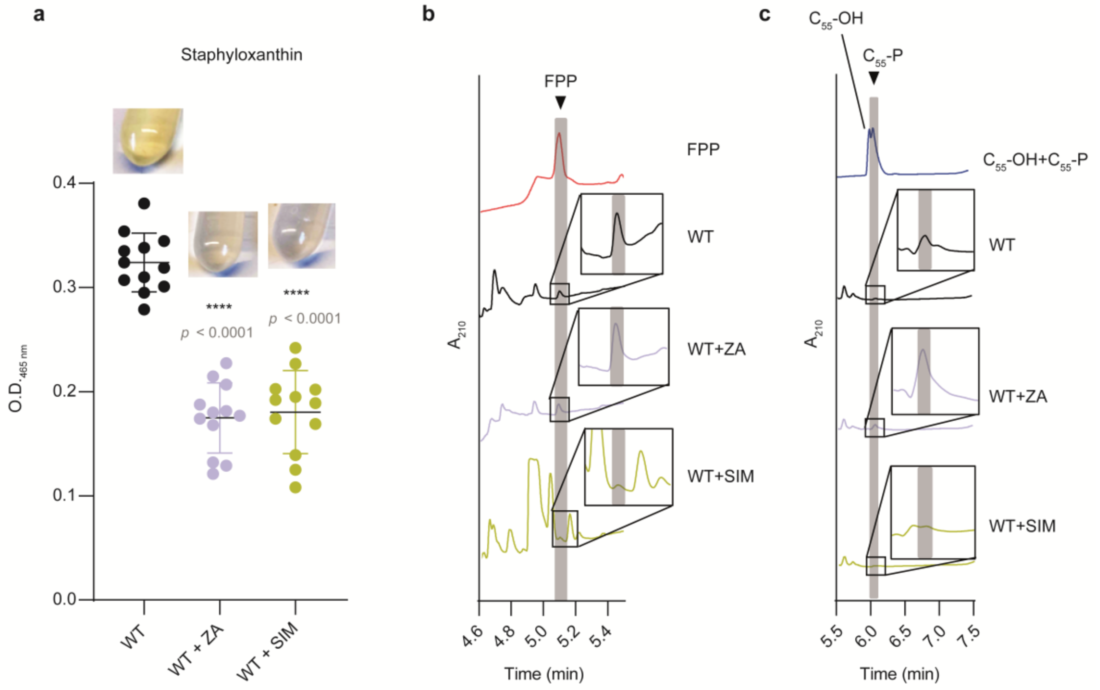

Supplementary Fig. 4. Effects of statins on staphyloxanthin and isoprenoid intermediates in the WT strain. (**a**) Staphyloxanthin levels in WT strain ± Simvastatin (SIM) and Zaragozic acid (ZA). Statistical significance is determined by one-way ANOVA (data are mean ± standard deviation from n = 12 independent biological replicates, with significance indicated as $*p < 0.05$; $**p < 0.01$; $***p < 0.001$; $****p < 0.0001$). (**b**) Representative UPLC-UV chromatograms (210 nm absorbance) showing farnesyl pyrophosphate (FPP) levels in WT strains with and without statin treatment. A 2 mM FPP standard was used as a control. Zoom-in views of the relevant peak are shown. (**c**) Representative UPLC-UV chromatograms (210 nm absorbance) illustrating undecaprenyl phosphate (C_{55}-P) levels in WT strain with and without statins. 200 nmol C_{55}-OH (undecaprenol) and C_{55}-P (undecaprenyl phosphate) standards were used as controls. Zoom-in views of the relevant peaks are shown. SIM: Simvastatin; ZA: Zaragozic acid. Source data are provided as a Source Data file.

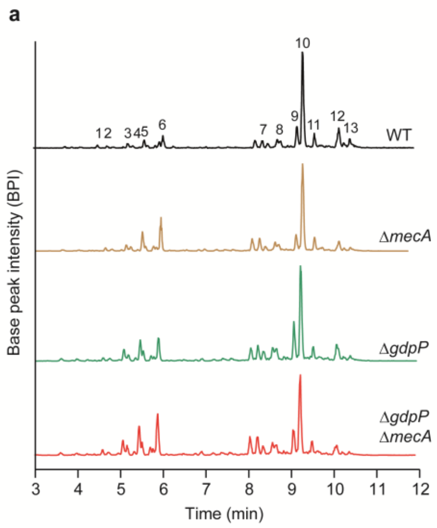

n.º	Identity
1	M4
2	M5
3	M4(G_5)
4	M4(G_6)
5	M5(G_1)
6	M5(G_5)
7	$^{O\text{-acet}}$D5(G_5)-4(G_4)
8	$^{O\text{-acet}}$D5(G_5)-4(G_5)
9	D5(G_5)-4(G_1)
10	D5(G_5)-4(G_5)
11	D5(G_5)-4(G_5)$^{\text{-non-NH}_2}$
12	T5(G_5)-4(G_5)-4(G_5)
13	T5(G_5)-4(G_5)-4(G_5)-4(G_5)

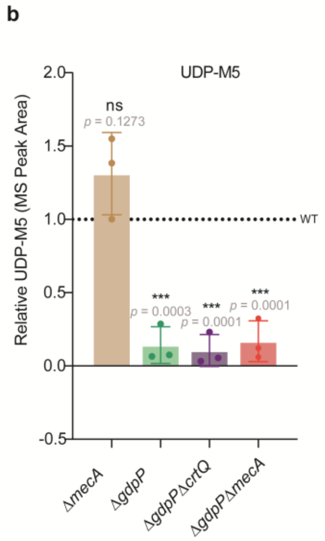

Supplementary Fig. 5. Impact of *mecA* and *gdpP* mutations on peptidoglycan metabolism and oxacillin susceptibility. (**a**) Representative base peak intensity (BPI) chromatograms from qualitative analysis of peptidoglycan in *mecA*, *gdpP*, and *gdpP-mecA* mutants compared to the WT strain (JE2). Detected muropeptides are numbered and annotated according to their identities as determined by LC–MS; details are provided in Supplementary Table 3. (**b**) Quantification of relative UDP-M5 MS peak area in *mecA*, *gdpP*, *gdpP-crtQ*, and *gdpP-mecA* mutant strains compared to the WT strain (n = 3). Data are mean ± standard deviation from n = 3 independent biological replicates. Statistical significance was assessed using a Student's t-test (unpaired, two-tailed) with significance indicated as *p < 0.05; **p < 0.01; ***p < 0.001; ****p < 0.0001; ns, not significant. Source data are provided as a Source Data file.

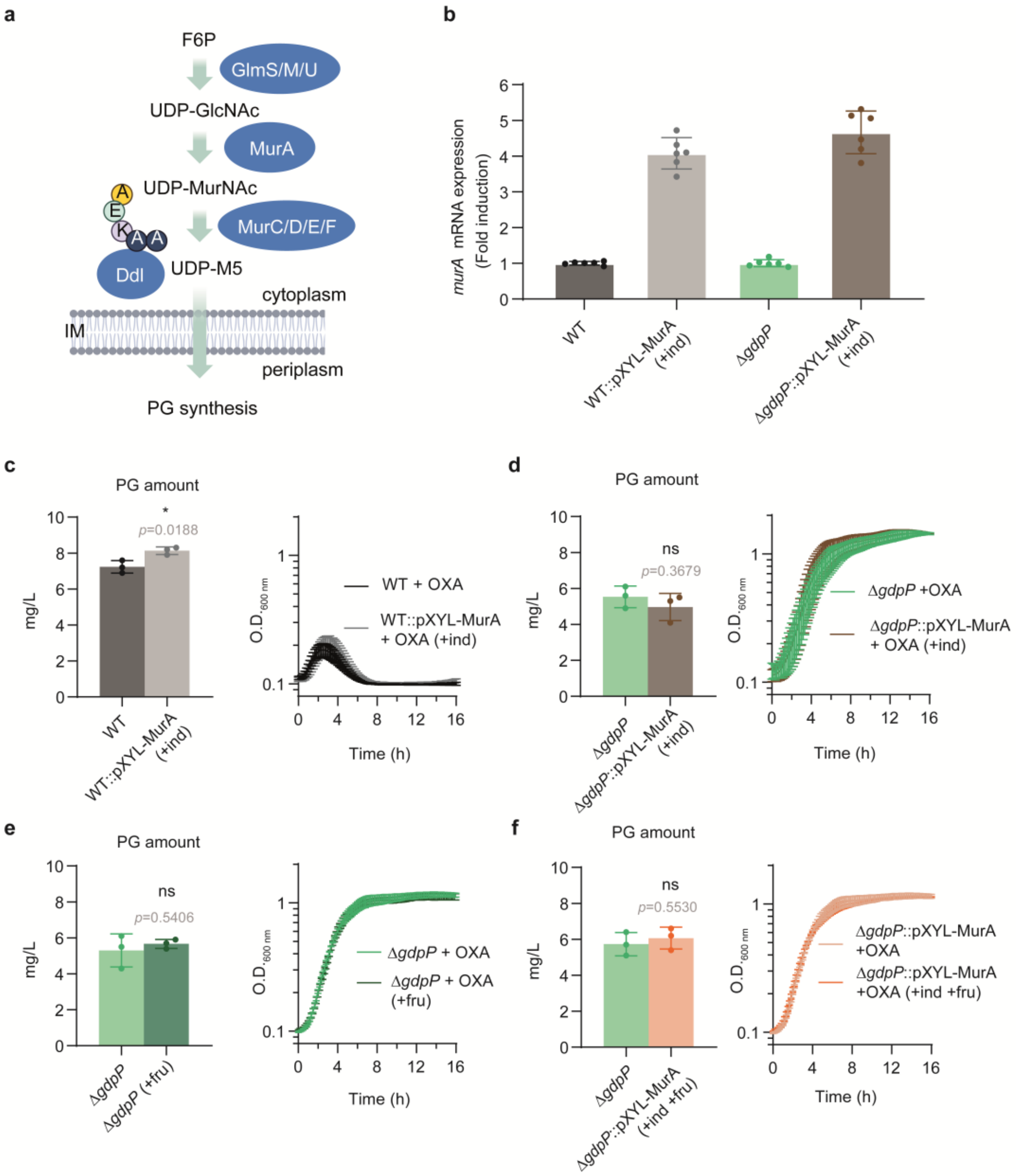

Supplementary Fig. 6. Effects of *gdpP* mutation and MurA overexpression on peptidoglycan synthesis and growth in *S. aureus:* (**a**) Schematic representation of the cytoplasmic precursor pathway for peptidoglycan (PG) synthesis, initiated from fructose-6-phosphate (F6P), with key enzymes such as MurA (UDP-N-acetylglucosamine 1-carboxyvinyltransferase) and Ddl (D-alanine–D-alanine ligase) being essential for the formation of UDP-MurNAc-pentapeptide (UDP-M5). (**b**) Relative mRNA expression levels of *murA* in the *gdpP* mutant compared to the WT strain, determined by qRT-PCR. Data are mean ± standard deviation from n = 6 independent biological replicates. (**c-d**) Quantification of peptidoglycan (PG) levels and growth curves

in the presence of oxacillin (16 µg/mL) for the WT strain, *gdpP* mutant, and both overexpressing MurA from the plasmid pAmyXYL under a xylose-inducible promoter. (**e-f**) Quantification of PG levels and growth curves of *gdpP* mutant, and *gdpP* mutant overexpressing MurA under treatment with oxacillin (16 µg/mL) ± D-(-)-Fructose. (+ind) = Xylose 10 mM; (+fru) = D-(-)-fructose 10 mM. PG quantification data are presented as mean ± standard deviation from $n = 3$ independent biological replicates. Growth-curve data are presented as mean ± standard deviation from $n = 4$ independent biological replicates. Statistical significance was conducted using a Student's *t*-test (unpaired, two-tailed) with significance indicated as *$p < 0.05$; **$p < 0.01$; ***$p < 0.001$; ****$p < 0.0001$; ns, not significant. Panel a was created in BioRender: https://BioRender.com/2x71gll. Source data are provided as a Source Data file.

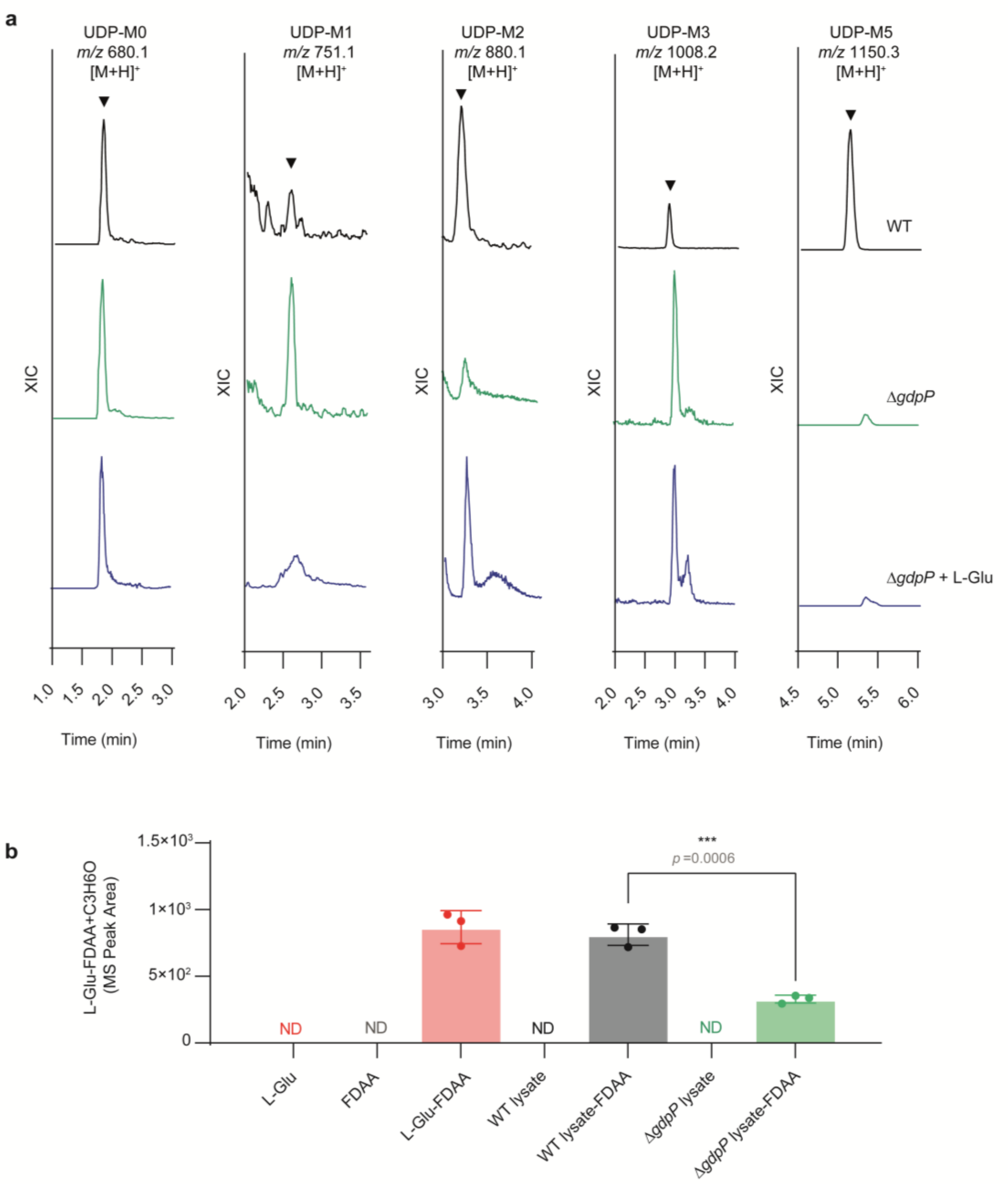

Supplementary Fig. 7. Metabolic profiling of UDP-MurNAc intermediates and L-glutamate. (**a**) Extracted ion chromatograms (XIC) comparing WT, *gdpP* mutant, and *gdpP* mutant supplemented with 10 mM L-glutamate (L-Glu). UDP-M0 (*m/z* 680.1 $[M+H]^+$), UDP-M1 (*m/z* 751.1 $[M+H]^+$), UDP-M2 (*m/z* 880.1 $[M+H]^+$), UDP-M3 (*m/z* 1008.2 $[M+H]^+$) and UDP-M5 (*m/z* 1150.3 $[M+H]^+$). (**b**) Derivatized L-Glu levels from WT and *gdpP* mutant lysates based on MS peak area. Data are mean ± standard deviation from n = 3 independent biological replicates. ND = Not detected. Statistical significance was conducted using a Student's *t*-test (unpaired, two-tailed) with significance indicated

as *$p < 0.05$; **$p < 0.01$; ***$p < 0.001$; ****$p < 0.0001$. Source data are provided as a Source Data file.

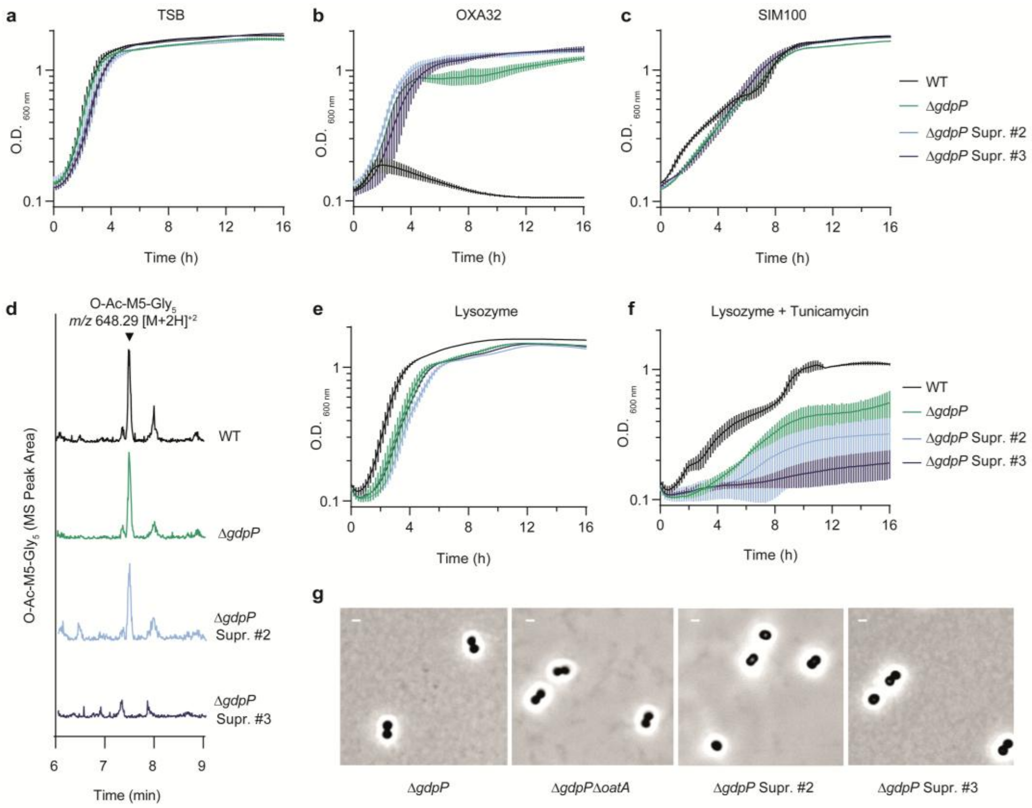

Supplementary Fig. 8. Growth of suppressor mutants, the parental strain, and WT under different conditions. (**a**) Growth curves in Tryptic Soy Broth (TSB), (**b**) oxacillin (OXA) at 32 µg/mL and (**c**) simvastatin (SIM) at 100 µM. (**d**) Representative extracted ion chromatograms (XIC) of M5-Gly_5 O-acetylated (*m/z* 648.29 $[M+2H]^{+2}$) obtained by LC-MS. (**e**) Growth curves in presence of lysozyme (4 mg/mL) and (**f**) tunicamycin (0.4 µg/mL) combined with lysozyme. (**g**) Representative microscopy images of the indicated strains (scale bar: 1 µm). For panels a-c, data are presented as mean ± standard deviation from *n* = 3 independent biological replicates, except for panels e-f, for which *n* = 4 independent biological replicates. Source data are provided as a Source Data file.

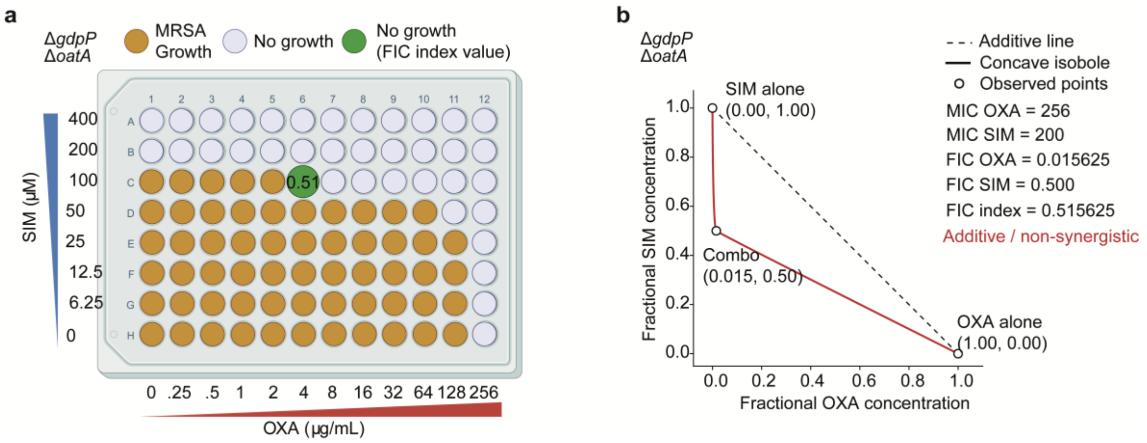

Supplementary Fig. 9. Checkerboard analysis of oxacillin and simvastatin interaction in the Δ*gdpP* Δ*oatA* strain. (a) Representative checkerboard assay using two-fold serial dilutions of oxacillin (OXA, 0–256 µg/mL) and simvastatin (SIM, 0–400 µM). Orange circles indicate visible growth, light-blue circles indicate no visible growth, and the green circle indicates the inhibitory drug combination used for fractional inhibitory concentration (FIC) calculation. **(b)** Isobologram corresponding to panel **a**. Drug concentrations are normalized to the MIC of each compound alone. Open circles indicate observed inhibitory points, the dashed line indicates the theoretical additive line, and the solid line indicates the fitted isobole. FIC index = 0.516. Data are mean ± standard deviation from *n* = 3 independent biological replicates. Panel a was created in BioRender: https://BioRender.com/99mpdfr. Source data are provided as a Source Data file.

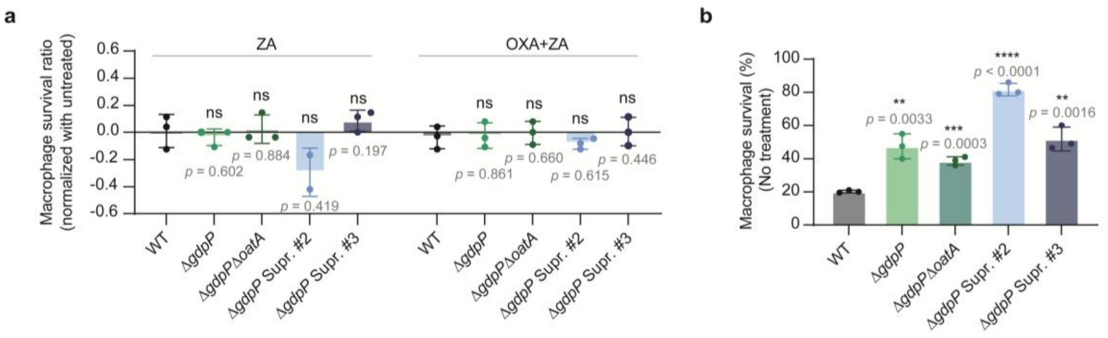

Supplementary Fig. 10. Influence of zaragozic acid and oxacillin on murine macrophage response to *S. aureus i*nfection. (**a**) Log_2 macrophage survival ratios normalized to the untreated condition, measured after infection of bone marrow–derived macrophages (BMDMs) at a 4:1 bacteria:macrophage ratio. Treatments compared include zaragozic acid (ZA, 25 µM) alone or in combination with oxacillin (OXA, 250 µg/mL). The strains tested included WT, suppressor mutants, and parental strains. Data are mean ± standard deviation from *n* = 3 independent biological replicates. Statistical analyses were performed using an unpaired *t*-test (two-tailed) with Welch's correction. ns, not significant. (**b**) Survival (%) of murine bone marrow–derived macrophages following in vitro infection at a 5:1 bacteria:macrophage ratio. Data are mean ± standard deviation from *n* = 3 independent biological replicates. Statistical significance was assessed using a Student's *t*-test (unpaired, two-tailed) with significance indicated as **p* < 0.05; ***p* < 0.01; ****p* < 0.001; *****p* < 0.0001, ns, not significant. Source data are provided as a Source Data file.

Supplementary Tables.

Supplementary Table 1. List of strains used in this study.

Bacteria	Genotype/use	Reference
Staphylococcus aureus MRSA (USA300 LAC)	Clinical isolate	[1]
Staphylococcus aureus MRSA (USA300 LAC)	*crtQ*::Tn	[1]
Staphylococcus aureus MRSA (USA300 LAC)	*mecA*::Tn	[1]
Staphylococcus aureus RN4220	Restriction-deficient mutant of strain 8325-4	[2]
Staphylococcus aureus USA300 JE2	Δ*mecA*	This study
Staphylococcus aureus USA300 JE2	Δ*crtQ*	This study
Staphylococcus aureus USA300 JE2	Δ*gdpP*	This study
Staphylococcus aureus USA300 JE2	Δ*gdpP* *amy*::pAmy-xyl- $gdpP_{WT}$	This study
Staphylococcus aureus USA300 JE2	Δ*gdpP* Δ*crtQ*	This study
Staphylococcus aureus USA300 JE2	Δ*gdpP* Δ*mecA*	This study
Staphylococcus aureus USA300 JE2	WT *amy*::pAmy-xyl-*murA*	This study
Staphylococcus aureus USA300 JE2	Δ*gdpP* amy::pAmy-xyl-*murA*	This study
Staphylococcus aureus USA300 JE2	Δ*gdpP* suppressor #1 rpoC* P431L	This study
Staphylococcus aureus USA300 JE2	Δ*gdpP* suppressor #2 *rpoC** V1160E	This study
Staphylococcus aureus USA300 JE2	Δ*gdpP* suppressor #3 *rpoC** V1160E; *oatA** G45fs	This study
Staphylococcus aureus USA300 JE2	Δ*gdpP* suppressor #4 *rpoB** G540D; *oatA** A564fs	This study
Staphylococcus aureus USA300 JE2	Δ*gdpP* Δ*oatA*	This study
S. pneumoniae D39	D39 ST581	[3]
S. pneumoniae D39	D39 ST581 Δ*pde1* Δ*pde2*	[3]
Escherichia coli DH5α	Used for cloning and plasmid production	[4]
Escherichia coli IM08	Used for plasmid purification to transform *S. aureus*	[5]

Supplementary Table 2. List of plasmids used in this study.

Plasmid	Description or use	Source/Ref
pMAD	Temperature-sensitive shuttle vector, employed for allelic replacement (gene deletion and functional analysis). *bla*, Ori pBR325, *ermC*, *bgaB*, *pclp*B	[6]
pMAD-Δ*mecA*	pMAD carrying upstream and downstream regions of *mecA* for allelic replacement	This study
pMAD-Δ*crtQ*	pMAD carrying upstream and downstream regions of *crtQ* for allelic replacement	This study
pMAD-Δ*gdpP*	pMAD carrying upstream and downstream regions of *gdpP* for allelic replacement	This study
pMAD-Δ*oatA*	pMAD carrying upstream and downstream regions of *oatA* for allelic replacement	This study
pAmy-XYL	pMAD based Integrative vector, carrying a xylose-inducible expression cassette, was used to insert an allele into the *amyE* locus of *S. aureus*, enabling inducible expression for phenotypic complementation.	[7]
pAmy-XYL-*murA*	pMAD-based integrative vector carrying a xylose-inducible promoter fused to the WT *murA* gene, used for insertion into the *amyE* locus of *S. aureus* to allow inducible expression for phenotypic complementation	This study
pAmy-XYL-*gdpP*	pMAD-based integrative vector carrying a xylose-inducible promoter fused to the WT *gdpP* gene, used for insertion into the *amyE* locus of *S. aureus* to allow inducible expression for phenotypic complementation	This study

Supplementary Table 3. Identified muropeptides.

Identity	Proposed Structure	Observed *m/z* value	Observed ion
M4	GlcNAc-MurNAc-L-Ala-D-Glu-L-Lys-D-Ala	897.44	$[M+H]^+$
M5	GlcNAc-MurNAc-L-Ala-D-Glu-L-Lys-D-Ala-D-Ala	968.48	$[M+H]^+$
M4(G_5)	GlcNAc-MurNAc-L-Ala-D-Glu-L-Lys(L-Gly_5)-D-Ala	1182.55	$[M+H]^+$
M4(G_6)	GlcNAc-MurNAc-L-Ala-D-Glu-L-Lys(L-Gly_2)-D-Ala(L-Gly_4)	1239.57	$[M+H]^+$
M5(G_1)	GlcNAc-MurNAc-L-Ala-D-Glu-L-Lys(L-Gly_1)-D-Ala-D-Ala	1025.50	$[M+H]^+$
$^{O\text{-}acet\text{-}}$M5(G_5)	$^{O\text{-}acet\text{-}}$M5(L-Gly_5)	648.29	$[M+2H]^{2+}$
M5(G_5)	GlcNAc-MurNAc-L-Ala-D-Glu-L-Lys(L-Gly_5)-D-Ala-D-Ala	1253.58	$[M+H]^+$
$^{O\text{-}acet\text{-}}$D5(G_5)-4(G_4)	$^{O\text{-}acet\text{-}}$M5(L-Gly_5)-M4(L-Gly_4)	783.02	$[M+3H]^{3+}$
$^{O\text{-}acet\text{-}}$D5(G_5)-4(G_5)	$^{O\text{-}acet\text{-}}$M5(L-Gly_5)-M4(L-Gly_5)	821.04	$[M+3H]^{3+}$
D5(G_5)-4(G_1)	M5(L-Gly_5)-M4(L-Gly_1)	730.68	$[M+3H]^{3+}$
D5(G_5)-4(G_5)	M5(L-Gly_5)-M4(L-Gly_5)	806.70	$[M+3H]^{3+}$
D5(G_5)-4(G_5)$^{\text{-non-}NH_2}$	M5(L-Gly_5)-M4(L-Gly_5) (non-amidated Glu)	807.03	$[M+3H]^{3+}$
T5(G_5)-4(G_5)-4(G_5)	M5(L-Gly_5)-M4(L-Gly_5)-M4(L-Gly_5)	1194.22	$[M+3H]^{3+}$
Tt5(G_5)-4(G_5)-4(G_5) -4(G_5)	M5(L-Gly_5)-M4(L-Gly_5)-M4(L-Gly_5)-M4(L-Gly_5)	1187.04	$[M+4H]^{4+}$
UDP-M0	UDP-MurNAc	680.1	$[M+H]^+$
UDP-M1	UDP-MurNAc-L-Ala	751.1	$[M+H]^+$
UDP-M2	UDP-MurNAc-L-Ala-D-Glu	880.1	$[M+H]^+$
UDP-M3	UDP-MurNAc-L-Ala-D-Glu-L-Lys	1008.2	$[M+H]^+$
UDP-M5	UDP-MurNAc-L-Ala-D-Glu-L-Lys-D-Ala-D-Ala	1150.3	$[M+H]^+$
Gly_5-Lipid II (delipidated)	(Und-PP)-GlcNAc-MurNAc-L-Ala-D-isoGln-L-Lys(L-Gly_5)-D-Ala-D-Ala	1331.51	$[M+H]^+$

M: Monomer; D: Dimer; T: trimers; Tt: tetramer; GlcNAc: N-acetylglucosamine; MurNAc: N-acetylmuramic acid (NAM); L/D-Ala: alanine; D-Glu: glutamic acid; L-Lys: lysine; L-Gly: glycine; UDP: Uridine-5'-diphosphate; Und-PP: undecaprenyl pyrophosphate; D-isoGln: D-iso-glutamine (γ-carboxyl group of D-Glu is amidated).

Supplementary References

1. Fey, P. D. *et al.* A genetic resource for rapid and comprehensive phenotype screening of nonessential *Staphylococcus aureus* genes. *mBio* **4**, e00537-12 (2013).
2. Novick, R. P. Genetic systems in staphylococci. *Methods Enzymol* **204**, 587–636 (1991).
3. Zarrella, T. M., Yang, J., Metzger, D. W. & Bai, G. Bacterial Second Messenger Cyclic di-AMP Modulates the Competence State in *Streptococcus pneumoniae*. *J Bacteriol* **202**, e00691-19 (2020).
4. Hanahan, D. Studies on transformation of *Escherichia coli* with plasmids. *J Mol Biol* **166**, 557–580 (1983).
5. Monk, I. R., Tree, J. J., Howden, B. P., Stinear, T. P. & Foster, T. J. Complete Bypass of Restriction Systems for Major *Staphylococcus aureus* Lineages. *mBio* **6**, e00308-00315 (2015).
6. Arnaud, M., Chastanet, A. & Débarbouillé, M. New vector for efficient allelic replacement in naturally nontransformable, low-GC-content, gram-positive bacteria. *Appl Environ Microbiol* **70**, 6887–6891 (2004).
7. Yepes, A., Koch, G., Waldvogel, A., Garcia-Betancur, J.-C. & Lopez, D. Reconstruction of mreB expression in *Staphylococcus aureus* via a collection of new integrative plasmids. *Appl Environ Microbiol* **80**, 3868–3878 (2014).
