# Supplementary material for: Statin-induced lipid carrier stress reveals a conserved vulnerability in β-lactam-resistant Gram-positive bacteria
Source: Nat Commun. 2026 Jul 20;17:6680. doi: 10.1038/s41467-026-75729-8 (PMC13385884; doi:10.1038/s41467-026-75729-8)
Supplement: Supplementary file 2 — Description of Additional Supplementary File [file 41467_2026_75729_MOESM2_ESM.pdf]

Description of the Additional Supplementary Files

Supplementary Data 1-

Contains the results of the laboratory evolution experiment.

Supplementary Data 2-

Contains the oligonucleotide sequences used in this study
