# Supplementary material for: Statin-induced lipid carrier stress reveals a conserved vulnerability in β-lactam-resistant Gram-positive bacteria
Source: Nat Commun. 2026 Jul 20;17:6680. doi: 10.1038/s41467-026-75729-8 (PMC13385884; doi:10.1038/s41467-026-75729-8)
Supplement: Supplementary file 5 — Reporting summary [file 41467_2026_75729_MOESM5_ESM.pdf]

nature portfolio

Corresponding author(s): Felipe Cava

Last updated by author(s): June 24, 2026

Reporting Summary

Nature Portfolio wishes to improve the reproducibility of the work that we publish. This form provides structure for consistency and transparency in reporting. For further information on Nature Portfolio policies, see our Editorial Policies and the Editorial Policy Checklist.

Statistics

For all statistical analyses, confirm that the following items are present in the figure legend, table legend, main text, or Methods section.

n/a	Confirmed	
☐	☒	The exact sample size (*n*) for each experimental group/condition, given as a discrete number and unit of measurement
☐	☒	A statement on whether measurements were taken from distinct samples or whether the same sample was measured repeatedly
☐	☒	The statistical test(s) used AND whether they are one- or two-sided<br>*Only common tests should be described solely by name; describe more complex techniques in the Methods section.*
☒	☐	A description of all covariates tested
☐	☒	A description of any assumptions or corrections, such as tests of normality and adjustment for multiple comparisons
☐	☒	A full description of the statistical parameters including central tendency (e.g. means) or other basic estimates (e.g. regression coefficient) AND variation (e.g. standard deviation) or associated estimates of uncertainty (e.g. confidence intervals)
☐	☒	For null hypothesis testing, the test statistic (e.g. *F*, *t*, *r*) with confidence intervals, effect sizes, degrees of freedom and *P* value noted<br>*Give P values as exact values whenever suitable.*
☒	☐	For Bayesian analysis, information on the choice of priors and Markov chain Monte Carlo settings
☒	☐	For hierarchical and complex designs, identification of the appropriate level for tests and full reporting of outcomes
☒	☐	Estimates of effect sizes (e.g. Cohen's *d*, Pearson's *r*), indicating how they were calculated

Our web collection on statistics for biologists contains articles on many of the points above.

Software and code

Policy information about availability of computer code

Data collection	No custom code was generated for this study. Optical density measurements were collected using Biotek Gen5 (v.08). Microscopy images were acquired with Zeiss Zen Blue Edition (v2.0.0.0). Liquid chromatography (LC) and mass spectrometry (LC-MS/MS) data were collected using Waters Empower 3.6, Waters UNIFI 1.8.1, and MassLynx 4.2. Sequencing data were generated using Illumina MiSeq.
Data analysis	No custom code was generated for this study. Optical density measurements and microscopy data were analyzed using GraphPad Prism (v8.0–9.0) and Fiji/ImageJ (v1.53) with the MicrobeJ plugin. Sequencing data generated by MiSeq were processed using BWA-MEM for read alignment, Picard tools for marking and removing duplicates, and FreeBayes and VarScan for SNP and indel detection. LC-MS/MS data were analyzed using Waters UNIFI (v1.8.1) and MassLynx (v4.2). Chemical structure prediction was performed using ACD/Labs ChemSketch. Phylogenetic analyses were performed to investigate evolutionary relationships among bacterial sequences. Multiple sequence alignments were conducted using Clustal Omega or T-Coffee Expresso, and phylogenetic trees were generated using PhyloT v2 and visualized with iTOL. Protein structure prediction and visualization were performed using AlphaFold (models retrieved from the AlphaFold Protein Structure Database) and UCSF ChimeraX (v1.10.1) for structural visualization and figure rendering. Isobolograms were generated and visualized in R using ggplot2.

For manuscripts utilizing custom algorithms or software that are central to the research but not yet described in published literature, software must be made available to editors and reviewers. We strongly encourage code deposition in a community repository (e.g. GitHub). See the Nature Portfolio guidelines for submitting code & software for further information.

Data

Policy information about availability of data

All manuscripts must include a data availability statement. This statement should provide the following information, where applicable:

- Accession codes, unique identifiers, or web links for publicly available datasets
- A description of any restrictions on data availability
- For clinical datasets or third party data, please ensure that the statement adheres to our policy

All data supporting the findings of this study are included in the paper, its supplementary information, and Source Data files, which will be provided with the manuscript. Whole genome sequencing (WGS) data generated in this study have been deposited at the European Nucleotide Archive (ENA) and are publicly accessible under accession number PRJEB108212. All other data generated in this study are available from the corresponding author upon reasonable request.

Research involving human participants, their data, or biological material

Policy information about studies with human participants or human data. See also policy information about sex, gender (identity/presentation), and sexual orientation and race, ethnicity and racism.

Reporting on sex and gender	N/A
Reporting on race, ethnicity, or other socially relevant groupings	N/A
Population characteristics	N/A
Recruitment	N/A
Ethics oversight	N/A

Note that full information on the approval of the study protocol must also be provided in the manuscript.

Field-specific reporting

Please select the one below that is the best fit for your research. If you are not sure, read the appropriate sections before making your selection.

☒ Life sciences ☐ Behavioural & social sciences ☐ Ecological, evolutionary & environmental sciences

For a reference copy of the document with all sections, see nature.com/documents/nr-reporting-summary-flat.pdf

Life sciences study design

All studies must disclose on these points even when the disclosure is negative.

Sample size	The sample size was not predetermined or calculated using formal statistical methods. Sample sizes were chosen following standard practices in molecular biology and microbiology, based on literature (e.g., PMID: 31289173) and variability observed in previous laboratory experience. In most experiments, at least three biological replicates were used to account for random variation, providing sufficient statistical power to rule out differences due to inherent biological variation. Larger sample sizes were used for measurements of bacterial width and length, as indicated in the corresponding figure legends.
Data exclusions	No data was excluded from the study and analyses.
Replication	Experiments described in the manuscript were fully replicated, with three or more biological replicates. Where chromatograms or microscopy images are shown, these are representative of three biological replicates.
Randomization	No specific randomization processes were necessary as the experimental outcome does not depend on the order in which samples were analyzed in the experiments. Appropriate controls were used in all assays. In all experiments, control and experimental groups were done in isogenic strains.
Blinding	Blinding was not performed as all measurements in the study are quantitative at defined timepoints, and knowing the order or identity of a sample does not affect the results.

Reporting for specific materials, systems and methods

We require information from authors about some types of materials, experimental systems and methods used in many studies. Here, indicate whether each material, system or method listed is relevant to your study. If you are not sure if a list item applies to your research, read the appropriate section before selecting a response.

Materials & experimental systems

n/a	Involved in the study
☒	☐ Antibodies
☐	☒ Eukaryotic cell lines
☒	☐ Palaeontology and archaeology
☐	☒ Animals and other organisms
☒	☐ Clinical data
☒	☐ Dual use research of concern
☒	☐ Plants

Methods

n/a	Involved in the study
☒	☐ ChIP-seq
☒	☐ Flow cytometry
☒	☐ MRI-based neuroimaging

Eukaryotic cell lines

Policy information about cell lines and Sex and Gender in Research

Cell line source(s)	Primary macrophages differentiated from bone marrow cells of BALB/c OlaHsd mice.
Authentication	N/A; primary cells were isolated fresh from mice.
Mycoplasma contamination	Cultures were maintained under sterile conditions; mycoplasma testing was not performed for these primary cells.
Commonly misidentified lines (See ICLAC register)	N/A; primary cells were directly isolated from animals and are not immortalized or commercially available cell lines.

Animals and other research organisms

Policy information about studies involving animals; ARRIVE guidelines recommended for reporting animal research, and Sex and Gender in Research

Laboratory animals	BALB/c OlaHsd mice (Mus musculus), 8–10 weeks old, 16–19 g body weight, obtained from Envigo. Mice were housed in polypropylene cages under standardized lighting conditions, with ad libitum access to food and water.
Wild animals	N/A
Reporting on sex	Both male and female mice were included in infection experiments.
Field-collected samples	N/A
Ethics oversight	All procedures were approved by the Regional Government of Madrid, Spain (license PROEX 049.3-24) and were conducted in accordance with Spanish legislation and EU Directive 2010/63/EU.

Note that full information on the approval of the study protocol must also be provided in the manuscript.

Plants

Seed stocks	N/A
Novel plant genotypes	N/A
Authentication	N/A
