# Supplementary material for: Statin-induced lipid carrier stress reveals a conserved vulnerability in β-lactam-resistant Gram-positive bacteria
Source: Nat Commun. 2026 Jul 20;17:6680. doi: 10.1038/s41467-026-75729-8 (PMC13385884; doi:10.1038/s41467-026-75729-8)
Supplement: Supplementary file 6 — Transparent Peer Review file [file 41467_2026_75729_MOESM6_ESM.pdf]

nature portfolio

Peer Review File

Statin-induced lipid carrier stress reveals a conserved vulnerability in β-lactam-resistant Gram-positive bacteria

Corresponding Author: Professor Felipe Cava

This file contains all reviewer reports in order by version, followed by all author rebuttals in order by version.

Version 0:

Reviewer comments:

Reviewer #1

(Remarks to the Author)

In this manuscript, the authors investigate the interaction between simvastatin (SIM) and oxacillin (OXA) in Staphylococcus aureus, focusing on the role of GdpP, a regulator of cyclic-di-AMP signaling. The study explores how ΔgdpP mutations influence the response to SIM–OXA combination treatment and identifies additional suppressor mutations that modulate this phenotype. The authors further examine the impact of GdpP loss on peptidoglycan precursor pools and cell wall metabolism, proposing that alterations in cell wall biosynthesis underlie the observed drug interactions.

The main significance of the manuscript could appear to be the potential use of SIM in combination with oxacillin. However, synergy between statins and OXA has already been reported. The more specific advance of the present study is therefore the analysis of how ΔgdpP mutations affect this interaction. This makes the scope of the work somewhat narrower, but the manuscript still provides substantial and interesting data on the effects of ΔgdpP mutations, as well as suppressor mutations, on peptidoglycan biosynthesis, including biochemical evidence that GdpP affects the PG precursor pool.

The study is experimentally thorough and carefully done, and the authors systematically test their main hypotheses and provide strong supporting biochemical data to prove or disprove them. The figures are particularly well prepared, with helpful schematics illustrating the experiments and affected pathways. One general presentation suggestion is to use more distinct colours in some graphs, especially in growth curves where several traces overlap (where for example black and dark blue can be difficult to distinguish in the graph labels)

Major comments

Line 59 – Efflux pumps are not a very relevant mechanism of resistance against beta-lactams, as these antibiotics do not have to cross the membrane to act. The reference 5 cited by the authors is a review that mentions the existence of efflux pumps in MRSA strains, but does not show that these efflux pumps are related to beta-lactam resistance. Authors can rephrase the sentence or cite a study directly showing an important role of efflux pumps in beta-lactam resistance.

Line 73 – To the best of my knowledge, there is no strong evidence that SSCmec, which is a chromosomal element, can be transferred by conjugation in natural settings. Reference #10 mentions in the introduction the possibility of conjugative plasmids having some role: “For large SCCmec structures, additional processes may be necessary to integrate into S. aureus, such as conjugative plasmids as a carrier or subsequent multiple recombination events.” But it is not a paper showing that conjugation is a mechanism for SCCmec transfer. Authors may provide an appropriate reference, or delete “conjugation” from the sentence.

Line 123 - RpoB/C SNPs are also common in strains resulting from LTEE in the crtQ mutant and in presence of ZA. This should be acknowledged, and the rationale for focusing the analysis primarily on GdpP SNPs should be explained.

Lines 128/129 and Suppl Tabel 1 – The SNP found in FtsK in the 4 independent lines is exactly the same (A665G). It seems unlikely that the identical nucleotide substitution would arise independently four times, which raises the possibility that this mutation was already present in a common ancestor, for example in the colony or plate used to initiate the cultures. Authors should discuss this possibility, or repeat the experiment to exclude it.

Lines 129-131 – Can authors clarify what they mean in this sentence?

Line 147 (and various other places in the manuscript) The tile of this section mentions “synthetic lethality of simvastatin and GdpP loss”. However, the lethality is conditional on the presence of oxacillin. The gdpP mutant still grows with SIM alone (although growth is impaired, so synthetic sickness is observed). Growth only stops with OXA+SIM. Therefore, statements throughout the manuscript referring to synthetic lethality between SIM and GdpP loss should be rephrased.

Line 192 – The increase in PG content in the WT strain upon MurA overexpression is modest. The wording should be adjusted to reflect that the effect is minor.

Line 243-245 – Authors mention that suppressor mutants have increased sensitivity to lysozyme, because of a frameshift mutation in OatA. However, this increased sensitivity is observed in Suppressors #2 and #3, but only #3 has the OatA mutation (Sup Fig 7b). In fact, in this fig, growth of Sup #2 in the presence of lysozyme is slight worse that that of #3. Can authors explain this or moderate the sentence?

Sup Fig 7b – A microscopy image of cells labelled for example with a membrane dye and shown larger in the figure, would be more informative.

Section starting on line 265 – The conclusion” Together, these results demonstrate that SIM can effectively restore antibiotic sensitivity in highly resistant gdpP mutants in vivo.” would be considerably stronger if data obtained in macrophages would be validated in the mouse model.

Line 301 – The statement “These two proteins are broadly distributed across the Bacillota phylum (formerly Firmicutes), suggesting a conserved functional link.” is overstated. Broad distribution of two proteins does not by itself imply a functional link. Please rephrase.

Lines 326-333 – The discussion of altered K+ levels and their phenotypic consequences should be clearly presented as a hypothesis based on prior literature, not as a conclusion from the present study, since intracellular K+ levels were not measured here. Also, the suggestion that glutamate is diverted into arginine as a compensatory cation is speculative and should not be presented as mechanistically established.

Quantification of PG amount – It is unclear how peptidoglycan amount is normalized in experiments such as Fig. 3b. From the Methods, it appears that PG is purified from the same culture volume at the same OD (0.5), and the dry mass of sacculi is then normalized to the initial OD. However, this approach does not account for differences in cell size. Since the gdpP mutant is smaller, sacculus mass per OD could vary because of differences in cell size, surface area, or purification recovery, and not necessarily because PG synthesis is impaired. Did the authors verify that the same OD corresponds to the same cell number in WT and ΔgdpP cultures?

Minor comments
Line 120-121 – The supplementary figure is referred to inconsistently as “Figure S1c” and “Supplementary Figure 1a–c.”

Line 124 – “Multiple conditions” is misleading here. GdpP mutations are found in two of the four conditions tested, and not in the absence of statins. It would be clearer to state explicitly which conditions yielded GdpP mutations.

Line 134 – The use of “however” suggests that previous work reported ΔgdpP mutants to have the same cell size as WT. If that is not the intended meaning, the sentence should be rephrased.

Figure 1f – colours used for OXA+ZA and OXA+SIM are very similar. Consider choosing colours that are easier to distinguish.

Line 159 – While – correct capital letter

Bibliography - Please check formatting throughout. For example, species names and gene names should be italicized where appropriate.

SI material - Line 97 – Sup Table S5 not S4 should be mentioned.

Reviewer #2

(Remarks to the Author)
It has previously been found that statins resensitize MRSA to beta-lactams by disrupting functional membrane microdomains (FMM), thereby compromising structuring of the beta-lactam resistant PBP2a. In this work, by searching for mechanisms that counteract the effect of statins, it is found that in particular the cyclic-di-AMP singalling is a critical factor regulating beta-lactam sensitivity. By quantifying peptidoglycan precursor / lipid carriers in DgdpP, it is found that these mutants have a dysbalanced precursor synthesis, pointing to peptidoglycan precursor / lipid carriers as a key vulnerability point in MRSA strain. Building on this, statin-induced peptidoglycan precursor / lipid carrier stress is shown to be a conserved vulnerability

across Gram-positive bacterial pathogens, which could be used as a treatment target.

Overall, the manuscript presents an impressive combination of genetic approaches, analyses and quantification of PG precursors / lipids, in vivo assay and cross-species studies, all presented in a coherent manner to support the conclusions in the manuscript. Of particular interest, new mechanistic insights into factors affecting the critical PG-precursor synthesis is provided as well as new insights into the cellular effects of c-di-AMP signalling in S. aureus. In addition, the work presents possible strategies to counteract resistance across Gram-positive bacteria. The manuscript is well written, and the data/figures are nicely presented. The methodology section is mostly described in sufficient detail (see below).

Major comment:
It is found that mutations in the in GdpP suppresses the effect of Zaragozic acid (ZA) and the crtQ-mutation. In line with this, deletion of gdpP increased MIC to oxacillin (also consistent previous reports). Interestingly, further assays demonstrate how lack of GdpP cause alterations/dysbalance in PG precursor levels. These observed effects are presumably caused by the increased c-di-AMP levels in the mutant cells (and not the lack of GdpP per se), however, this is not explicitly demonstrated. It would be interesting to further verify that the effect is a result of c-di-AMP levels, either by quantifying c-di-AMP levels and by testing whether other genetic alterations resulting in altered c-di-AMP levels have phenotypic effects consistent with DgdpP (e.g. overexpression of DacA should have similar effect as DgdpP, while GdpP overexpression should have the opposite effect).

Minor comments:
- Supplementary figure 1. In addition to the gdpP mutation. It would be of interest if the discussion on these other mutations could be included.
- Line 128. “Instead, SIM drove resistance via mutations in ftsK, suggesting a distinct adaptive trajectory (Suppl. Figure 1d). This divergence implies that SIM interferes with a pathway that functionally intersects with GdpP’s role in resistance, rather than simply selecting for compensatory mutations.” The reasoning in these sentences is fully clear. Please elaborate or consider rephrasing.
- Line 381. Refer to the source of the S. pneumoniae D39-strain.
- Line 588. Please include more details in the description of how PG amounts were determined (including volumes, details on the freeze-drying).

Reviewer #3

(Remarks to the Author)
In this manuscript by Torrens et al., the authors explore the relationship between susceptibility to beta-lactam antibiotics and the effect of statins in S. aureus. Through efficient and effective use of multiple molecular microbiology techniques, the authors identify several key enzymes that influence peptidoglycan biosynthesis and are responsible for the interaction between beta-lactam and statin treatment. Furthermore, they demonstrate the potential for a broader application of their work by showing similar effects from statins in a parallel system in S. pneumoniae. The authors’ focus on not only an important human pathogen but also clinically relevant medications makes this very impactful work. Overall, the paper is very well written with logical, well thought out experiments that use a variety of genetic, phenotypic, and biochemical approaches to address their hypotheses. As such, I found very minimal issues with the paper.

I do think that the paper could benefit from better characterizing the relationship/interactions between oxacillin, simvastatin, and the genetic mutations they investigate with regards to growth inhibition. While the authors do present some MICs and growth curves under certain conditions throughout the paper, various different concentrations of antibiotics and statins are used in different experiments. Including data from checkboard assays looking at the interaction of oxacillin and simvastatin for the WT, crtQ::Tn, ΔgdpP, and ΔgdpP ΔoatA strains would be useful. Also related, is there a MIC for simvastatin alone? The paper does suggest a growth effect from simvastatin, but it is not clear at what concentration it could inhibit growth by itself, and whether there are differences in that value between the mutants.

Otherwise, I have a few minor points:

Line 56-57, this statement is inaccurate, there are still many therapeutic options for almost all MRSA isolates and the reason that surgical removal of infected tissue is often required is due to characteristics of S. aureus infections themselves. Recommendations regarding surgical removal is dependent on the type and nature of the S. aureus infection and not the resistance pattern of the isolate.

Supplementary Table 2- should be “Staphylococcus aureus RN4220” with a zero and not the letter O

Version 1:

Reviewer comments:

Reviewer #2

(Remarks to the Author)
The authors have sufficiently addressed the points raised in the review.

Reviewer #3

(Remarks to the Author)
All of my original concerns have been addressed in the revision and I have not further comments.

Open Access This Peer Review File is licensed under a Creative Commons Attribution 4.0 International License, which permits use, sharing, adaptation, distribution and reproduction in any medium or format, as long as you give appropriate credit to the original author(s) and the source, provide a link to the Creative Commons license, and indicate if changes were made.
In cases where reviewers are anonymous, credit should be given to 'Anonymous Referee' and the source.
The images or other third party material in this Peer Review File are included in the article's Creative Commons license, unless indicated otherwise in a credit line to the material. If material is not included in the article's Creative Commons license and your intended use is not permitted by statutory regulation or exceeds the permitted use, you will need to obtain permission directly from the copyright holder.
To view a copy of this license, visit https://creativecommons.org/licenses/by/4.0/

REVIEWER COMMENTS

Reviewer #1 (Remarks to the Author):

In this manuscript, the authors investigate the interaction between simvastatin (SIM) and oxacillin (OXA) in *Staphylococcus aureus*, focusing on the role of GdpP, a regulator of cyclic-di-AMP signaling. The study explores how Δ*gdpP* mutations influence the response to SIM–OXA combination treatment and identifies additional suppressor mutations that modulate this phenotype. The authors further examine the impact of GdpP loss on peptidoglycan precursor pools and cell wall metabolism, proposing that alterations in cell wall biosynthesis underlie the observed drug interactions. The main significance of the manuscript could appear to be the potential use of SIM in combination with oxacillin. However, synergy between statins and OXA has already been reported. The more specific advance of the present study is therefore the analysis of how Δ*gdpP* mutations affect this interaction. This makes the scope of the work somewhat narrower, but the manuscript still provides substantial and interesting data on the effects of Δ*gdpP* mutations, as well as suppressor mutations, on peptidoglycan biosynthesis, including biochemical evidence that GdpP affects the PG precursor pool. The study is experimentally thorough and carefully done, and the authors systematically test their main hypotheses and provide strong supporting biochemical data to prove or disprove them. The figures are particularly well prepared, with helpful schematics illustrating the experiments and affected pathways. One general presentation suggestion is to use more distinct colours in some graphs, especially in growth curves where several traces overlap (where for example black and dark blue can be difficult to distinguish in the graph labels).

We thank the reviewer for the positive assessment of our work. We have addressed all the points raised and improved the colours used.

Major comments

Line 59 – Efflux pumps are not a very relevant mechanism of resistance against beta-lactams, as these antibiotics do not have to cross the membrane to act. The reference 5 cited by the authors is a review that mentions the existence of efflux pumps in MRSA strains, but does not show that these efflux pumps are related to beta-lactam resistance. Authors can rephrase the sentence or cite a study directly showing an important role of efflux pumps in beta-lactam resistance.

We agree with the reviewer and have revised the sentence accordingly, removing the reference.

Line 73 – To the best of my knowledge, there is no strong evidence that SSCmec, which is a chromosomal element, can be transferred by conjugation in natural settings. Reference #10 mentions in the introduction the possibility of conjugative plasmids having some role: “For large SCCmec structures, additional processes may be necessary to integrate into *S. aureus*, such as conjugative plasmids as a carrier or subsequent multiple recombination events.” But it is not a paper showing that conjugation is a mechanism for SCCmec transfer. Authors may provide an appropriate reference, or delete “conjugation” from the sentence.

We thank the reviewer for this important point. We agree that direct evidence for SCCmec transfer by conjugation under natural conditions remains limited. However, experimental studies have shown that SCCmec can be captured by conjugative plasmids and transferred between staphylococci. To reflect this more accurately, we have revised the text to indicate that conjugation represents an experimentally supported mechanism that may operate under specific conditions, rather than a broadly established route of transfer in nature. The sentence now reads: "*The mecA gene is carried on a mobile genetic element known as the staphylococcal chromosomal cassette mec (SCCmec), which can be transferred between bacteria via transduction and, under specific conditions, through plasmid-mediated conjugation, contributing to the spread of MRSA and β-lactam resistance.*"

Line 123 - RpoB/C SNPs are also common in strains resulting from LTEE in the crtQ mutant and in presence of ZA. This should be acknowledged, and the rationale for focusing the analysis primarily on GdpP SNPs should be explained.

We thank the reviewer for this point and agree that *rpoB/rpoC* mutations are recurrent in LTEE, particularly in *crtQ* mutants and under ZA exposure. We have now explicitly acknowledged this and clarified our rationale for focusing on *gdpP*. In brief, *gdpP* mutations were repeatedly selected across independent lineages under conditions where oxacillin resistance was restored despite impaired membrane microdomain or staphyloxanthin function, and often arose in the absence of accompanying *rpoB/rpoC* changes, indicating that they can independently drive the resistance phenotype. In contrast, *rpoB/rpoC* mutations are common outcomes of long-term evolution experiments and typically reflect broader transcriptional or growth-related adaptation. We therefore prioritized *gdpP* as a more specific and mechanistically informative entry point into the pathways underlying resistance in this context. This rationale is now incorporated in the revised manuscript (revised manuscript lines 136–140).

Lines 128/129 and Suppl Tabel 1 – The SNP found in FtsK in the 4 independent lines is exactly the same (A665G). It seems unlikely that the identical nucleotide substitution would arise independently four times, which raises the possibility that this mutation was already present in a common ancestor, for example in the colony or plate used to initiate the cultures. Authors should discuss this possibility, or repeat the experiment to exclude it.

We thank the reviewer for this insightful observation. We agree that the recurrence of the identical *ftsK* A665G substitution across four independent lineages raises the possibility that this variant arose early during the experiment or was already present in the founding population. Furthermore, given its conservative nature (Ala→Gly) and location in the C-terminal region of FtsK, we cannot confidently assign a functional role to this substitution in the adaptation to SIM/OXA conditions. We have now explicitly acknowledged this possibility in the revised the Results section (revised manuscript lines 130–135) to avoid overinterpreting its contribution.

Lines 129-131 – Can authors clarify what they mean in this sentence?

We thank the reviewer for pointing out that this sentence was unclear. Our intention was to indicate that, unlike ZA or *crtQ* impairment, SIM did not select *gdpP* mutations, suggesting that SIM imposes a distinct selective constraint and disfavors the GdpP/c-di-AMP resistance route. We have simplified the text to clarify this point.

Line 147 (and various other places in the manuscript) The tile of this section mentions "synthetic lethality of simvastatin and GdpP loss". However, the lethality is conditional on the presence of oxacillin. The gdpP mutant still grows with SIM alone (although growth is impaired, so synthetic sickness is observed). Growth only stops with OXA+SIM. Therefore, statements throughout the manuscript referring to synthetic lethality between SIM and GdpP loss should be rephrased.

We agree that the lethality is conditional on oxacillin treatment. We have therefore revised the section title and corresponding statements throughout the manuscript to avoid implying that simvastatin and GdpP loss are lethal in the absence of β-lactam stress. Specifically, we now refer to this interaction as oxacillin-dependent synthetic lethality, while distinguishing it from the growth impairment observed with simvastatin alone, which is more appropriately described as synthetic sickness in the Δ*gdpP* mutant.

Line 192 – The increase in PG content in the WT strain upon MurA overexpression is modest. The wording should be adjusted to reflect that the effect is minor.

We have edited the sentence to indicate that the change is modest but significant.

Line 243-245 – Authors mention that suppressor mutants have increased sensitivity to lysozyme, because of a frameshift mutation in OatA. However, this increased sensitivity is observed in Suppressors #2 and #3, but only #3 has the OatA mutation (Sup Fig 7b). In fact, in this fig, growth of Sup #2 in the presence of lysozyme is slight worse that that of #3. Can authors explain this or moderate the sentence?

We thank the reviewer for this observation. We agree that sensitivity to lysozyme alone is not strictly associated with *oatA* status, as Suppressor #2 also shows reduced growth despite carrying a wild-type *oatA* allele. We have therefore revised the text to clarify that the *oatA*-associated phenotype is most evident under combined lysozyme and tunicamycin stress, where suppressors carrying *oatA* mutations show increased sensitivity compared with the strain bearing only the *rpoC* mutation. Accordingly, we now describe this as an *oatA*-linked increase in sensitivity under combined stress conditions rather than attributing the effect solely to lysozyme. This is consistent with previous reports showing that teichoic acid defects sensitize *S. aureus* to lysozyme, particularly in combination with altered PG O-acetylation (revised manuscript References 32 and 35).

Sup Fig 7b – A microscopy image of cells labelled for example with a membrane dye and shown larger in the figure, would be more informative.

We thank the reviewer for this suggestion and agree that the original images were too small. As no clear morphological differences were observed between the strains, we opted not to introduce additional staining. Instead, we have provided enlarged views of the existing images to improve clarity.

Section starting on line 265 – The conclusion" Together, these results demonstrate that SIM can effectively restore antibiotic sensitivity in highly resistant gdpP mutants in vivo." would be considerably stronger if data obtained in macrophages would be validated in the mouse model.

We agree that validating simvastatin-mediated resensitization in a mouse model would strengthen this point. However, in the intranasal infection model, Δ*gdpP* and suppressor strains already show a strong fitness defect, with markedly reduced lung colonization compared with wild type. Under these conditions, host clearance dominates, making it difficult to detect any additional effect of simvastatin and oxacillin. For this reason, we used macrophage infection assays to test resensitization under more controlled conditions. The mouse model was instead used to address whether *gdpP* mutants and suppressor strains can sustain infection in vivo, which they fail to do.

We have revised the conclusion (revised manuscript lines 312–313) to clearly separate these points, distinguishing macrophage-based evidence for resensitization from the in vivo fitness defect observed in the mouse model.

Line 301 – The statement "These two proteins are broadly distributed across the Bacillota phylum (formerly Firmicutes), suggesting a conserved functional link." is overstated. Broad distribution of two proteins does not by itself imply a functional link. Please rephrase.

We have rephrased the sentente as follows: "*These two proteins are broadly distributed across the Bacillota phylum (formerly Firmicutes), motivating us to test whether the genetic interaction observed in MRSA extends to other species*".

Lines 326-333 – The discussion of altered K+ levels and their phenotypic consequences should be clearly presented as a hypothesis based on prior literature, not as a conclusion from the present study, since intracellular K+ levels were not measured here. Also, the suggestion that glutamate is diverted into arginine as a compensatory cation is speculative and should not be presented as mechanistically established.

We thank the reviewer for this point. We agree that these interpretations should be framed as hypotheses rather than conclusions. We have revised the paragraph accordingly, clearly distinguishing literature-based mechanisms from our observations and presenting this as a working model linking K^+ homeostasis, glutamate metabolism, and PG precursor availability.

The new edited paragraph reads: "*Mechanistically, we propose a double bottleneck centered on soluble PG precursors. First, prior work shows that elevated c-di-AMP in gdpP mutants impairs K^+ import*[28,39–41]*, which can diminish Ddl activity*[29] *and lead to UDP-M3 accumulation—a model consistent with our*

observations. Second, based on literature, K^+ limitation perturbs the glutamate–glutamine pool[42–44]; we hypothesize that this may favour diversion of glutamate toward arginine biosynthesis as a compensatory positively charged osmolyte[45], which would compromise MurD-dependent UDP-M2 synthesis[46] and further limit PG precursor availability. In line with this model, we observed significantly lower L-glutamate in gdpP lysates versus WT (Figure 4c, 8, Suppl. Figure 7b)."

Quantification of PG amount – It is unclear how peptidoglycan amount is normalized in experiments such as Fig. 3b. From the Methods, it appears that PG is purified from the same culture volume at the same OD (0.5), and the dry mass of sacculi is then normalized to the initial OD. However, this approach does not account for differences in cell size. Since the gdpP mutant is smaller, sacculus mass per OD could vary because of differences in cell size, surface area, or purification recovery, and not necessarily because PG synthesis is impaired. Did the authors verify that the same OD corresponds to the same cell number in WT and ΔgdpP cultures?

We agree that differences in cell size and in the relationship between OD and cell number are important considerations when interpreting PG yield. To address this, we determined viable cell counts from matched aliquots of the same mid-log phase cultures used for PG purification. As expected, cultures of the Δ*gdpP* mutant contained more cells than wild type at the same OD, consistent with its smaller cell size. For example, at OD=0.5, wild-type cultures contained approximately 2.9–3.3 × 10exp8 CFU/mL, whereas Δ*gdpP* cultures contained approximately 4.5–5.1 × 10exp8 CFU/mL.

We therefore normalized PG yield to cell number using paired cultures according to: CFU-corrected PG amount = raw PG amount × (WT CFU/mL ÷ sample CFU/mL), with wild-type values assigned a correction factor of 1. After this correction, Δ*gdpP* still showed reduced PG levels compared with wild type.

While CFU does not directly report total biomass or cell envelope content, this approach accounts for the major difference in cell number at a given OD. The persistence of the phenotype after normalization therefore supports that the reduced PG levels reflect a genuine decrease in PG accumulation rather than an artifact of differences in cell size or OD-to-cell-number relationships.

We have revised the Methods to clarify this normalization procedure and added the corresponding OD values, raw PG yields, CFU/mL measurements, correction factors, and normalized values to the source data file for the PG quantification figures.

Minor comments

Line 120-121 – The supplementary figure is referred to inconsistently as "Figure S1c" and "Supplementary Figure 1a–c."

We have fixed this inconsistency.

Line 124 – "Multiple conditions" is misleading here. GdpP mutations are found in

two of the four conditions tested, and not in the absence of statins. It would be clearer to state explicitly which conditions yielded GdpP mutations.

The conditions that promote or prevent *gdpP* mutations in OXA are specified in the next sentence (revised manuscript lines 136-140). We think that reiterating them here would be redundant, and we aimed to avoid unnecessary repetition.

Line 134 – The use of “however” suggests that previous work reported Δ*gdpP* mutants to have the same cell size as WT. If that is not the intended meaning, the sentence should be rephrased.

We have removed “however”.

Figure 1f – colours used for OXA+ZA and OXA+SIM are very similar. Consider choosing colours that are easier to distinguish.

We have changed the colours to improved figures clarity.

Line 159 – While – correct capital letter

Fixed.

Bibliography - Please check formatting throughout. For example, species names and gene names should be italicized where appropriate.

Fixed.

SI material - Line 97 – Sup Table S5 not S4 should be mentioned.

Fixed.

Reviewer #2 (Remarks to the Author):

It has previously been found that statins resensitize MRSA to beta-lactams by disrupting functional membrane microdomains (FMM), thereby compromising structuring of the beta-lactam resistant PBP2a. In this work, by searching for mechanisms that counteract the effect of statins, it is found that in particular the cyclic-di-AMP singalling is a critical factor regulating beta-lactam sensitivity. By quantifying peptidoglycan precursor / lipid carriers in DgdpP, it is found that these mutants have a dysbalanced precursor synthesis, pointing to peptidoglycan precursor / lipid carriers as a key vulnerability point in MRSA strain. Building on this, statin-induced peptidoglycan precursor / lipid carrier stress is shown to be a conserved vulnerability across Gram-positive bacterial pathogens, which could be used as a treatment target.

Overall, the manuscript presents an impressive combination of genetic approaches, analyses and quantification of PG precursors / lipids, in vivo assay and cross-species studies, all presented in a coherent manner to support the conclusions in the manuscript. Of particular interest, new mechanistic insights into factors affecting the critical PG-precursor synthesis is provided as well as new insights into the cellular effects of c-di-AMP signalling in S. aureus. In addition, the work presents possible strategies to counteract resistance across

Gram-positive bacteria. The manuscript is well written, and the data/figures are nicely presented. The methodology section is mostly described in sufficient detail (see below).

We thank the reviewer for this positive and constructive evaluation and for recognizing the mechanistic insights, experimental breadth, and clarity of presentation provided by the study.

Major comment:

It is found that mutations in the in GdpP suppresses the effect of Zaragozic acid (ZA) and the *crtQ*-mutation. In line with this, deletion of gdpP increased MIC to oxacillin (also consistent previous reports). Interestingly, further assays demonstrate how lack of GdpP cause alterations/dysbalance in PG precursor levels. These observed effects are presumably caused by the increased c-di-AMP levels in the mutant cells (and not the lack of GdpP per se), however, this is not explicitly demonstrated. It would be interesting to further verify that the effect is a result of c-di-AMP levels, either by quantifying c-di-AMP levels and by testing whether other genetic alterations resulting in altered c-di-AMP levels have phenotypic effects consistent with DgdpP (e.g. overexpression of DacA should have similar effect as DgdpP, while GdpP overexpression should have the opposite effect).

We agree with the reviewer that the observed phenotypes are best explained by altered cyclic-di-AMP homeostasis rather than the absence of GdpP per se. Importantly, this interpretation is already supported by multiple lines of evidence presented in the manuscript.

First, our data in *S. pneumoniae* provide GdpP-independent support for this model. This organism lacks GdpP but encodes alternative c-di-AMP phosphodiesterases (Pde1 and Pde2). Consistent with previous work showing that the *pde1 pde2* double mutant accumulates elevated c-di-AMP (revised manuscript Reference 38), we find that this strain phenocopies *Staphylococcus aureus* Δ*gdpP*, including increased β-lactam resistance, sensitization to combined β-lactam and simvastatin treatment, and reduced UDP-MurNAc-pentapeptide levels (Fig. 7c–e).

Second, our evolution experiments indicate that the phenotype is linked to altered GdpP activity rather than complete loss of the protein. Across independent lineages, the majority of *gdpP* mutations are missense substitutions rather than truncations. We functionally characterized one such variant (*gdpP* V489E), which maps to the predicted phosphodiesterase domain (Fig. R1a and 1c), and found that it fully phenocopies Δ*gdpP*, including increased oxacillin resistance, simvastatin-dependent sensitization, and reduced PG precursor levels (Fig. R1b–e). These results indicate that partial impairment of GdpP function is sufficient to drive the phenotype.

In addition, following the reviewer's suggestion, we examined strains with altered c-di-AMP synthesis (*dacA* perturbations, (revised manuscript Reference 41)) and observed reciprocal effects, with reduced oxacillin resistance and restoration of UDP-MurNAc-pentapeptide levels, consistent with decreased c-di-AMP (Fig. R1d–e).

Together, these findings consistently link increased c-di-AMP levels to β-lactam resistance and PG precursor imbalance. The additional data further support this interpretation; however, as the evidence already included in the manuscript is sufficient to support this conclusion, we prefer not to incorporate these additional experiments to avoid unnecessary expansion. We have clarified this point in the text (revised manuscript lines 127-129).

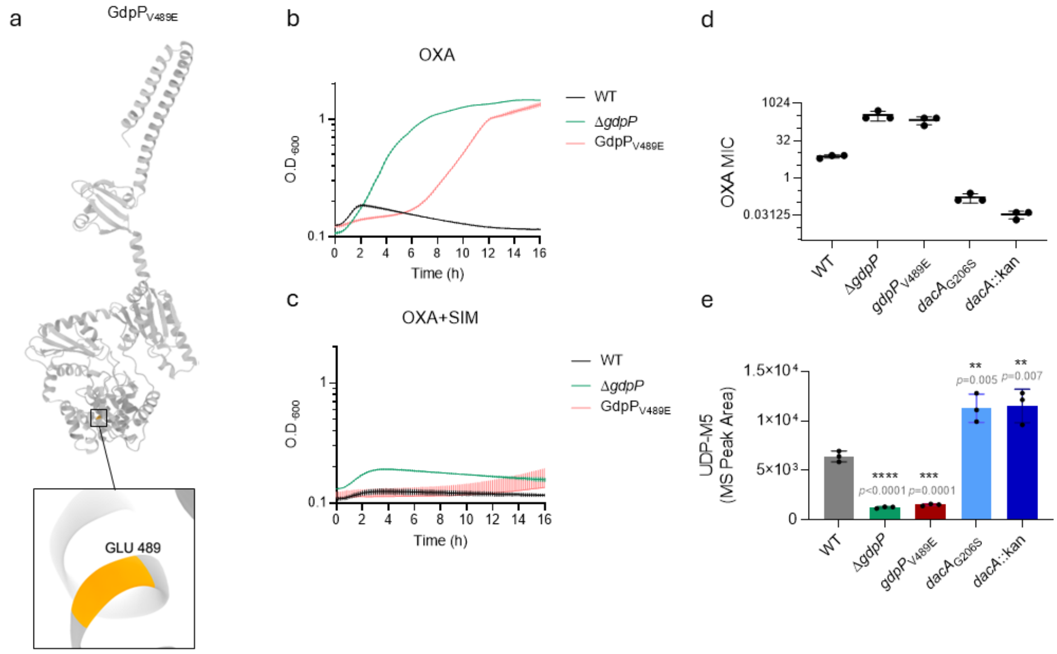

Figure R1. *gdpP*$_{V489E}$ phenocopies Δ*gdpP*, whereas DacA impairment produces reciprocal effects on oxacillin resistance and UDP-M5 levels. (a) Predicted structure of *S. aureus* GdpP showing the position of the evolved V489E substitution identified in the LTEE. The enlarged inset highlights residue 489 within the GdpP structure. **(b–c)** Growth curves of WT, Δ*gdpP*, and *gdpP*V$_{489E}$ strains in the presence of oxacillin alone (OXA) or oxacillin plus simvastatin (OXA + SIM). **(d)** Oxacillin MICs of WT, Δ*gdpP*, *gdpP*$_{V489E}$, *dacA*$_{G206S}$, and *dacA*::kan strains. **(e)** UDP-MurNAc-pentapeptide (UDP-M5) abundance determined by LC–MS and expressed as MS peak area in WT, Δ*gdpP*, *gdpP*$_{V489E}$, *dacA*$_{G206S}$, and *dacA*::kan strains. Statistical significance was determined relative to WT using an unpaired two-tailed Student's *t*-test. Data are presented as mean ± SD.

Minor comments:

- Supplementary figure 1. In addition to the gdpP mutation. It would be of interest if the discussion on these other mutations could be included.

We thank the reviewer for this suggestion. We have added a brief statement in the Results (Supplementary Fig. 1c; WGS data) to acknowledge additional recurrent or potentially relevant mutations, including variants in *norC*, *apt*, and *clpX* (revised manuscript lines 119-123). To maintain the focus of the study, we do not analyse each mutation in detail, but we clarify that these may represent additional adaptive responses to β-lactam/statin selection whose individual contributions remain to be defined.

- Line 128. "Instead, SIM drove resistance via mutations in ftsK, suggesting a distinct adaptive trajectory (Suppl. Figure 1d). This divergence implies that SIM interferes with a pathway that functionally intersects with GdpP's role in

resistance, rather than simply selecting for compensatory mutations." The reasoning in these sentences is fully clear. Please elaborate or consider rephrasing.

We thank the reviewer for this comment and apologise for the lack of clarity. Our original intention was to convey that the absence of *gdpP* mutations under SIM selection, together with the occurrence of alternative variants such as *ftsK*, suggests that SIM redirects adaptation away from the c-di-AMP/GdpP axis. However, as noted by Reviewer 1, the recurrent *ftsK* mutation may represent an early or low-impact variant.

We have therefore revised the text to avoid overinterpreting specific mutations and instead refer more broadly to SIM-associated variants (Supplementary Fig. 1d), clarifying that SIM induces a distinct adaptive trajectory without attributing a defined mechanistic role to individual SNPs (revised manuscript lines 130–135).

- Line 381. Refer to the source of the S. pneumoniae D39-strain.

We have indicated the source now.

- Line 588. Please include more details in the description of how PG amounts were determined (including volumes, details on the freeze-drying).

We have expanded the details in the methods section.

Reviewer #3 (Remarks to the Author):

In this manuscript by Torrens et al., the authors explore the relationship between susceptibility to beta-lactam antibiotics and the effect of statins in S. aureus. Through efficient and effective use of multiple molecular microbiology techniques, the authors identify several key enzymes that influence peptidoglycan biosynthesis and are responsible for the interaction between beta-lactam and statin treatment. Furthermore, they demonstrate the potential for a broader application of their work by showing similar effects from statins in a parallel system in S. pneumoniae. The authors' focus on not only an important human pathogen but also clinically relevant medications makes this very impactful work. Overall, the paper is very well written with logical, well thought out experiments that use a variety of genetic, phenotypic, and biochemical approaches to address their hypotheses. As such, I found very minimal issues with the paper.

We thank the reviewer for their positive assessment. We appreciate the constructive feedback and have addressed the minor points raised in the revised manuscript.

I do think that the paper could benefit from better characterizing the relationship/interactions between oxacillin, simvastatin, and the genetic mutations they investigate with regards to growth inhibition. While the authors do present some MICs and growth curves under certain conditions throughout the paper, various different concentrations of antibiotics and statins are used in different experiments. Including data from checkboard assays looking at the interaction of oxacillin and simvastatin for the WT, crtQ::Tn, ΔgdpP, and ΔgdpP

∆oatA strains would be useful. Also related, is there a MIC for simvastatin alone? The paper does suggest a growth effect from simvastatin, but it is not clear at what concentration it could inhibit growth by itself, and whether there are differences in that value between the mutants.

We thank the reviewer for this suggestion and agree that a more systematic analysis of the interaction between oxacillin and simvastatin across the relevant genetic backgrounds strengthens the study. To address this, we performed checkerboard assays using a consistent concentration matrix of oxacillin and simvastatin in WT, *crtQ*::Tn, Δ*gdpP*, and Δ*gdpP* Δ*oatA* strains (Fig. R2 a-d and S9 a-d in the revised manuscript).

These assays showed that simvastatin alone inhibits growth only at relatively high concentrations, with a MIC of ~200 μM under our conditions across all strains. Thus, the concentrations at which simvastatin potentiates oxacillin are well below those required for growth inhibition as a single agent.

The checkerboard analysis further revealed synergistic interactions between oxacillin and simvastatin in WT, *crtQ*::Tn, and Δ*gdpP* strains, with FIC index values of 0.375, 0.375, and 0.1875, respectively (Fig. R2 a-c and S3 a-c in the revised manuscript). The strongest interaction was observed in Δ*gdpP*, consistent with our model that loss of GdpP function creates a specific vulnerability to simvastatin-mediated lipid carrier stress during β-lactam treatment. In contrast, the Δ*gdpP* Δ*oatA* strain showed a higher FIC index (0.5156), consistent with an additive or weak interaction (Fig. R2d and S3 d in the revised manuscript) suggesting partial suppression of the combined effect.

We have revised the manuscript to include these data, clarify the simvastatin MIC, and provide a consistent quantitative framework for comparing oxacillin–simvastatin interactions across genetic backgrounds (revised manuscript lines 151–156 and 278–279; methods lines 464–477).

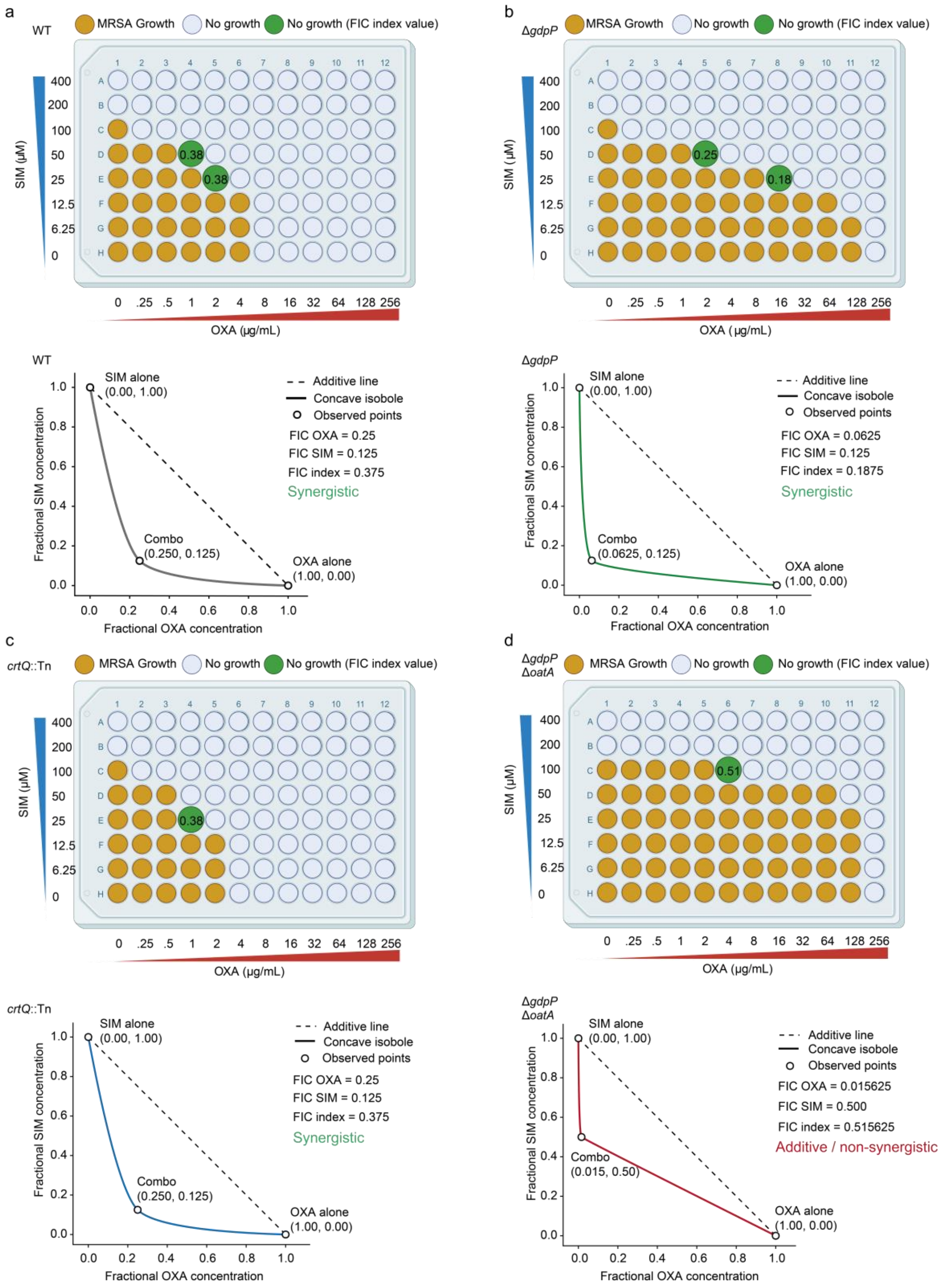

Figure R2. Checkerboard and isobologram analysis of SIM–OXA interactions. Each panel shows the checkerboard assay and corresponding normalized isobologram for (A) WT, (B) Δ*gdpP*, (C) *crtQ*::Tn, and (D) Δ*gdpP* Δ*oatA*. In checkerboards, orange wells indicate MRSA growth, blue wells indicate no growth, and green wells indicate the selected inhibitory combination used for FIC calculation. MICs were defined as the lowest concentrations preventing visible growth. In isobolograms, the MICs of SIM and OXA alone were normalized to (0, 1) and (1, 0), respectively. The dashed diagonal line represents theoretical additivity, the curved isobole shows the SIM–OXA interaction profile, and open circles indicate observed points. FIC indices

were calculated as FIC_OXA + FIC_SIM; synergy was defined as FIC index ≤ 0.5, additive/non-synergistic interaction as >0.5 to ≤1.0.

Otherwise, I have a few ***minor points***:

Line 56-57, this statement is inaccurate, there are still many therapeutic options for almost all MRSA isolates and the reason that surgical removal of infected tissue is often required is due to characteristics of S. aureus infections themselves. Recommendations regarding surgical removal is dependent on the type and nature of the S. aureus infection and not the resistance pattern of the isolate.

We thank the reviewer for this important clarification. We have revised the sentence to avoid implying that surgical intervention is a direct consequence of antibiotic resistance or that treatment options for MRSA are limited. The sentence now reads: "*Although most MRSA infections remain treatable, antimicrobial resistance complicates therapy and, particularly in invasive or deep-seated infections, effective management often requires surgical removal of infected tissue in addition to antibiotic treatment*"

Supplementary Table 2- should be "Staphylococcus aureus RN4220" with a zero and not the letter O

Fixed.
